# Effects of Probiotic Therapy on Periodontal and Peri-implant Treatments: An Umbrella Review

**DOI:** 10.1177/23800844241240474

**Published:** 2024-11-07

**Authors:** C. Mendonça, D. Marques, J. Silveira, J. Marques, R.F. de Souza, A. Mata

**Affiliations:** 1 Faculty of Dental Medicine, University of Lisbon, Lisbon, Portugal; 2 Biology and Oral Biochemistry Group, LIBPhys-FCT UIDB/04559/2020, Faculty of Dental Medicine, University of Lisbon, Lisbon, Portugal; 3 Center for Evidence-Based Dental Medicine, Faculty of Dental Medicine, University of Lisbon, Lisbon, Portugal; 4 Hugo Madeira Clinic—Advanced Aesthetics and Implantology, Lisbon, Portugal; 5 Institute of Implantology, Lisbon, Portugal; 6 Faculty of Dental Medicine and Oral Health Sciences, Montreal, QC, Canada

**Keywords:** periodontal diseases, periodontitis, peri-implantitis, microbiota, probiotics, synbiotics

## Abstract

**Introduction:**

The effectiveness of nonsurgical periodontal treatment is related to patient- and tooth-related factors. To overcome the limitations of the conventional approach, probiotics are one of the adjunct therapies that have been studied.

**Objectives:**

This umbrella review answered the focused question: in adult patients with periodontal diseases or peri-implant diseases, does the use of probiotic therapy as an adjuvant to nonsurgical periodontal treatment when compared with nonsurgical periodontal treatment alone affect treatment effectiveness and clinical disease parameters?

**Methods:**

A systematic electronic search to identify systematic reviews according to PICOS criteria, defined a priori, was used, and 5 electronic databases were searched (Medline, LILACS, Cochrane Central Registry of Controlled Trials, Google Scholar, and DANS EASY). Included systematic reviews were rated using quality assessment tools by 2 independent reviewers.

**Results:**

Thirty systematic reviews were identified evaluating the effectiveness of probiotics in periodontal and peri-implant disease treatment. A quantitative analysis of the results was not possible due to the high heterogeneity of clinical data. Seventeen of 31 reviews reported clinically relevant benefits of probiotic therapy as an adjuvant to scaling and root planning. Twenty-two reviews had a low risk of bias, 7 had a moderate risk, and 2 had a high risk.

**Conclusion:**

The evidence from the available studies is conflicting, which means that no definitive conclusions can be made about the effectiveness of probiotic therapy as an adjuvant to nonsurgical periodontal treatment. High-quality primary research studies are needed that control for known confounding variables.

**Knowledge Transfer Statement:**

This umbrella review provides some evidence regarding the efficacy of probiotics as an adjunct to nonsurgical periodontal therapy, despite some equivocal findings. However, short-term probiotic use alongside therapy appears to be advantageous; there is currently no evidence supporting their long-term benefits. We have also identified that probiotic research is primarily constrained by its origins in gastrointestinal applications, resulting in a lack of approved probiotics for dental use. This review highlights the need for extensive clinical research to ascertain their effectiveness in the oral environment. Nevertheless, the utilization of probiotics alongside periodontal treatment seems safe, with no reported adverse effects in patients. Thus, further clinical validations in oral health care settings are crucial.

## Introduction

According to the World Health Organization, periodontal diseases are a major public oral health problem that affects systemic health (Salas and Palacios 2010; Seminario-Amez et al. 2017; Al-Nasser and Lamster 2020). The global prevalence of this disease is around 90% (Barboza et al. 2020), with its peak incidence in the fourth decade of life (Al-Nasser and Lamster 2020). Although periodontal diseases are not life-threatening, they can have a considerable effect on morbidity (Al-Nasser and Lamster 2020).

Previous studies have demonstrated that periodontal diseases have a polymicrobial etiology, but this by itself is not sufficient for disease development (Salas and Palacios 2010; Mdala et al. 2013). Periodontal diseases are caused by the interaction of the triad of the host, microorganisms, and environmental factors, which culminates in dysbiosis of the oral cavity (Salas and Palacios 2010; Donos et al. 2020).

The main objective of periodontal disease treatment is to reduce the burden of pathogenic microorganisms, thus restoring the symbiotic flora around tooth or implant surfaces (Suvan 2005; Tomasi et al. 2007; Martin-Cabezas et al. 2016; Matsubara et al. 2016; Donos et al. 2020). Scaling and root planing (SRP) are regarded as the primary therapeutic approach to achieve this in dental substrates (Martin-Cabezas et al. 2016). Local debridement, implant-surface decontamination, and anti-infective therapies are the conventional procedures for implant substrates (Arbildo-Vega et al. 2021). Despite the effectiveness of nonsurgical periodontal treatment (NSPT), its response varies between and within patients (Hung and Douglass 2002; Tomasi et al. 2007), depending on patient and tooth factors, such as deep probing depths, inaccessible root furcation, and interproximal areas of malposed teeth (Tomasi et al. 2007; Martin-Cabezas et al. 2016). For peri-implantitis, NSPT alone seems insufficient to restore peri-implant tissues, which in most cases needs surgical regenerative approaches (Chala et al. 2020).

Several adjuvant therapies have been developed to overcome the limitations complementing the conventional approach (Donos et al. 2020), including probiotics. Probiotics are defined by the World Health Organization and by the Food and Agriculture Organization of the United States as “live microorganisms which, when administered in adequate amounts confer a health benefit on the host”. (http://www.who.int/foodsafety/fs_management/en/probiotic_guidelines.pdf) (Teughels et al. 2008; Salas and Palacios 2010; Twetman and Keller 2012; Zarco et al. 2012; Laleman and Teughels 2015; Seminario-Amez et al. 2017). Probiotics purpose aims to restore the balance of the oral microbial ecological environment by increasing the proportion of beneficial bacteria through competitive inhibition of periodontal pathogens, thus modulating the subsequent host response (Caglar et al. 2005; Marcotte et al. 2006; Stamatova and Meurman 2009; Salas and Palacios 2010; Yanine et al. 2013).

The efficacy of probiotic therapy remains inconclusive due to the multitude of species and subspecies, as well as varying administration protocols. Systematic reviews (SRs) investigating probiotics as adjuncts to nonsurgical periodontal treatment yield inconsistent results: some support their use, while others deem it clinically irrelevant. Given the complexity of clinical decisions, grounded in high-quality evidence, understanding the relative risks and benefits of probiotic therapy is crucial. Clinical decisions should rely on secondary or tertiary evidence. An umbrella review systematically compiles evidence, consolidating it into an accessible document. It assesses topics with competing interventions, focusing on existing systematic reviews to provide practical recommendations, identify gaps, and guide future research, aiming to analyze existing knowledge and improve clinical practice.

For that purpose, this umbrella review aimed to answer the following focused question: in adult patients with periodontal diseases or peri-implant diseases, does the use of probiotic therapy as an adjuvant to nonsurgical periodontal treatment when compared with nonsurgical periodontal treatment alone affect the treatment effectiveness and clinical disease parameters?

## Methods

### Protocol Registration and Reporting Format

This umbrella review was reported according to the Preferred Reporting Items for Systematic Reviews and Meta-Analyses (PRISMA) guidelines (Liberati et al. 2009) and previously registered in the International Prospective Register of Ongoing Systematic Reviews (PROSPERO; trial CRD42021254469).

### Eligibility Criteria

Table 1 shows the main inclusion criteria for the PICOS question, including primary and secondary outcomes. We define probiotic therapy as the administration of a probiotic species either alone or in combination. There were no publication time or country restrictions; only systematic reviews published in English, Portuguese, Spanish, or French were included The exclusion criteria were as follows: 1) methodology not compatible with the systematic review according to the Cochrane Handbook for Systematic Reviews of Interventions definition (Higgins and Green 2011), 2) evaluating illnesses other than periodontal diseases, 3) including healthy periodontal patients, and 4) evaluating therapies different from probiotics.

**Table 1.** Components of PICOS Question.

		PICOS Question
**P**	Patients	Adult patients (≥18 y) diagnosed with periodontal disease and/or peri-implant disease, according to the 1999 Armitage classification of periodontal diseases and conditions
**I**	Intervention or exposure	Probiotic therapy alone or a combination of species (*Lactobacillus*, *Bifidobacterium*, *Streptococcus*, *Bacillus*, *Clostridium*, *Saccharomyces*, *Pediococcus*, and subspecies of each)
**C**	Comparison	Conventional therapies (nonsurgical treatment, subgingival debridement, manual mechanical therapy, scaling and root planning) alone or with placebo
**O**	Outcomes	**Primary outcomes:** improvement on clinical parameters (such as PPD, CAL, BOP, bone loss around teeth or implants, survival rate of implants, tooth loss) **Secondary outcomes:** the influence of systemic diseases and microbiological analysis
**S**	Study design and duration	Systematic reviews of randomized control trials or nonrandomized trials

BOP, bleeding on probing; CAL, clinical attachment level; PPD, periodontal probing depth.

### Information Sources and Searches

Five electronic databases were searched up to December 2022: Medline (via PubMed), LILACS, Cochrane Central Registry of Controlled Trials (CENTRAL), Google Scholar (first 300 references), and a database listing of unpublished studies (DANS EASY Archive available at https://doi.org/10.17026/dans-xtf-47w5). Detailed search strategies were adopted, combined with screening manual reference lists and contacting corresponding authors via email to ask about additional research work on the subject or knowledge of any accessible ongoing projects. The search strategy is presented in Appendix 2.

### Study Selection and Data Extraction

Two reviewers independently screened titles and abstracts (when available) for eligibility based on the eligibility criteria and recorded detailed reasons for excluding studies. Full-text reports were obtained and reviewed for the included SRs and those with insufficient information in the title and abstract to make a clear decision. Two reviewers independently extracted data from the included SRs according to a predetermined datasheet form for systematic reviews from the Joanna Briggs Institute (JBI). The corresponding authors of potentially relevant articles or articles with data that needed further clarification were contacted via email and asked about missing data, additional research work on the subject, or if they were aware of any accessible ongoing projects.

Disagreements between the reviewers about the study selection and data collection were resolved through discussion until reaching a consensus. If necessary, a third reviewer was involved.

### Risk of Bias in Individual Studies

The JBI Critical Appraisal tools for use in the JBI Systematic Reviews—Checklist for Systematic Reviews were applied to assess the methodologic quality of the included SRs. Two reviewers independently assessed the included SRs and scored each question (yes, unclear, no, not applicable). Any discrepancies were discussed until reaching a consensus. Cohen’s κ coefficients and asymptotic standard errors were used to evaluate the interrater agreement for individual questions and the overall score, considering the following κ interpretation: poor agreement, <0; slight agreement, 0.0 to 0.20; fair agreement, 0.21 to 0.40; moderate agreement, 0.41 to 0.60; substantial agreement, 0.61 to 0.80; and almost perfect agreement, 0.81 to 1.00 (Landis and Koch 1977). The final score of each SR was calculated based on the percentage of positive answers (yes) only. Each study’s risk of bias was subsequently categorized according to the final score as high (≤49%), moderate (50–69%), or low (≥70%) (Saletta et al. 2019).

### Data Analysis and Synthesis of the Results

The included SRs were qualitatively synthesized, and summary tables were created with the measured effect for every SR or individual results from each SR successively. The results were categorized according to the specific outcome (primary and secondary) as clinically relevant (favors probiotic therapy) versus not clinically relevant (does not favor probiotic therapy) and separated by substrate (tooth or implant).

## Results

### Study Selection

Appendix 3 shows a flow diagram of the article screening process for inclusion in the review. The combined electronic search identified 2,660 articles, with 53 articles included for full-text review. In turn 2, 23 studies were excluded for not meeting the inclusion criteria (Appendix 4). Two additional records were identified through a manual search of the relevant studies’ references, resulting in 30 SRs accepted for qualitative evaluation, 1 unpublished work, and 1 protocol.

### Study Characteristics

Table 2 summarizes the characteristics of the included SRs (Teughels et al. 2011; Yanine et al. 2013; Gruner et al. 2016; Jayaram et al. 2016; Martin-Cabezas et al. 2016; Matsubara et al. 2016; Priyanka et al. 2016; Seminario-Amez et al. 2017; Ikram et al. 2018; Akram et al. 2020; Barboza et al. 2020; Barootchi et al. 2020; Donos et al. 2020; Gao et al. 2020; Ho et al. 2020; Silva et al. 2020; Song and Liu 2020; Vives-Soler and Chimenos-Kustner 2020; Abdulkareem et al. 2021; Arbildo-Vega et al. 2021; Canut-Delgado et al. 2021; Corbella et al. 2021; Hu et al. 2021; Mishra et al. 2021; Saïz et al. 2021; Gheisary et al. 2022; Hardan et al. 2022; Liu et al. 2022; Ng et al. 2022; Sayardoust et al. 2022). Twenty-two SRs assessed adjuvant probiotic therapy in periodontitis treatment (Teughels et al. 2011; Yanine et al. 2013; Gruner et al. 2016; Jayaram et al. 2016; Martin-Cabezas et al. 2016; Matsubara et al. 2016; Priyanka et al. 2016; Seminario-Amez et al. 2017; Ikram et al. 2018; Donos et al. 2020; Ho et al. 2020; Song and Liu 2020; Vives-Soler and Chimenos-Kustner 2020; Abdulkareem et al. 2021; Canut-Delgado et al. 2021; Corbella et al. 2021; Hu et al. 2021; Mishra et al. 2021; Gheisary et al. 2022; Hardan et al. 2022; Ng et al. 2022), with 6 SRs in gingivitis treatment (Yanine et al. 2013; Jayaram et al. 2016; Priyanka et al. 2016; Akram et al. 2020; Barboza et al. 2020; Liu et al. 2022). Five SRs evaluated peri-implantitis treatment (Barootchi et al. 2020; Gao et al. 2020; Silva et al. 2020; Arbildo-Vega et al. 2021; Sayardoust et al. 2022) and 1 SR in oral health treatment in general (related to periodontal disease) (Saïz et al. 2021).

**Table 2.** Main Characteristics of Included Systematic Reviews.

Author (Year)	Objectives	Participants	Description of Interventions/Phenomena of Interest	Range (y) of Included Details	Number of Studies Included	Types of Studies Included	Country of Origin of Included Studies	Analysis	Outcome Assessed	Significance/Direction
Silva et al. (2020)	The aim of this study was to determine the effects of probiotics on peri-implant diseases.	Patients diagnosed with peri implantitis or peri-implant mucositis	The use of probiotics as an adjuvant therapy on the nonsurgical treatment of peri-implant diseases	2015 to 2019	5	RCTs	Not reported	Qualitative analysis	Primary outcome: changes in BOP and PPD Secondary outcome: variables PI, microbiological and immunological parameters	Favors placebo
Abdulkareem et al. (2021)	Therefore, the aim of this review was to summarize the available literature, determining efficacy of using antibiotics during periodontal therapy and the effectiveness of alternative methods.	287 (probiotics)	One of the arms received SD with adjunctive antimicrobial or photodynamic therapy or probiotics. The other arm (control) should receive SD alone.	2013 to 2021	8	RCTs	Not reported	Qualitative analysis	Microbiological and clinical outcomes	Does not favor probiotics
Vives-Soler and Chimenos-Kustner (2020)	This systematic review aimed to assess the literature for the effectiveness of different probiotic strains as adjuvants to nonsurgical periodontal therapy.	326 participants	To compare combined manual therapy and probiotics or manual therapy and placebo	2010 to 2016	9	RCTs	Not reported	Narrative data analysis	Primary outcome: reduction in pocket probing depth Secondary outcomes: bleeding on probing, plaque index reduction and bacteria counts	Favors probiotics
Arbildo-Vega et al. (2021)	The aim of this article is to determine, through a systematic review and meta-analysis, the clinical effectiveness of LR in the treatment of PD.	Overall, there were 119 men and 153 women. All studies included patients of 18 y of age or older. The total number of patients treated and implants examined was 272.	The use of probiotics with LR in the treatment peri-implant disease	2016 to 2019	6	RCTs	Belgium, Saudi Arabia, Spain, Japan, and Sweden	Qualitative analysis and quantitative analysis	Probing depth reduction, change in plaque index, change in bleeding index	Favors probiotics
Song and Liu (2020)	Our meta-analysis aims at evaluating the magnitude of improvement in clinical and microbiological parameters, with administration of *Lactobacillus reuteri* alone in adjunct to SRP.	398 patients Patients with chronic periodontitis with 4 mm of attachment loss and pocket depth of 4 mm demanding nonsurgical periodontal therapy	Probiotics containing LR administered orally as lozenges, tablets, mouthwashes, toothpastes, chewing gums, and so on in adjunct to nonsurgical periodontal therapy	2010 to 2019	11	RCTs	Not reported	Qualitative analysis and quantitative analysis	Primary outcome: gain in CAL, reduction in PPD, and reduction in microbial levels	Favors probiotics
Hu et al. (2021)	To evaluate the efficacy of probiotics as an adjunctive therapy to SRP in the management of periodontitis.	919 participants Adult patient (age >18) was diagnosed with periodontitis.	The studies aimed to compare probiotic + SRP with placebo + SRP or SRP alone; the trials reported the primary clinical outcome, such as PPD, CAL gain, BOP, GI, and PI.	2010 to 2020	25	RCTs	Brazil, Sweden, Chile, China, India, Turkey, Belgium, Iran, Germany, Barcelona, Pakistan, Italy, Canada, Slovenia, Saudi Arabia, and Egypt	Qualitative synthesis and meta-analysis	Primary outcome: CAL, PPD, and BOP	Favors probiotic
Gruner et al. (2016)	We aimed to appraise trials assessing probiotics for managing caries and periodontal disease.	3,247 participants Dentate humans who consumed oral probiotics, regardless of the way of consumption or the probiotic species	We included randomized controlled trials comparing the efficacy of probiotics versus (placebo) control with regards to SM, LB, periodontal pathogen numbers, gingivitis, oral hygiene, caries incidence/experience increment, or periodontitis.	2001 to 2015	50	RCTs	Not reported	Meta-analysis and trial-sequential analysis	Bacterial numbers, GI, PI, BOP, PPD, CAL, and caries experience prevalence	Does not favor probiotics
Barboza et al. (2020)	This systematic review aimed to analyze the effects of probiotics on experimental gingivitis in humans.	181 participants Human adults presenting experimental gingivitis	Use of probiotic therapy	2009 to 2017	5	RCTs	Not reported	Qualitative analysis	Primary outcomes: gingivitis identified and graded by BOP, PI, and GI Secondary outcomes: the inflammatory response determined by GCF volume and biomarkers	Favors probiotics
Ng et al. (2022)	To comprehensively investigate the efficacy of adjunctive probiotics compared to placebo, using conventional and novel treatment outcomes.	Systemically healthy adults (18 y old) diagnosed with periodontitis	The intervention group consisted of patients receiving nonsurgical therapy with adjunctive probiotic therapy. No restrictions were placed on type of probiotic or method of administration. The comparator group consisted of patients receiving nonsurgical therapy with a placebo.	Supplementary materials (paid)	10	RCTs	Turkey, Chile, Brazil, India, and Hong Kong	Qualitative analysis and meta-analysis	Primary outcomes: percentage change of the total number of deeper sites (5 mm, 6 mm, 7 mm) before and after therapy Secondary outcomes: change in mean pocket probing depth (mm), percentage of patients in need of additional therapy, risk for disease progression, and microbiological and immunological results	Favors probiotics
Gou et al. (2020)	This systematic review and meta-analysis aimed to unveil whether adjunctive use of probiotics had additional clinical efficacy in the treatment of periodontitis.	376 participants Adults who were diagnosed with periodontitis and were systemically healthy and nonsmoking at any age range	Oral probiotic administration compared with placebo or without any interventions. Randomized controlled clinical trials were included when they 1) tested 1 or more probiotic agents as an adjunct to SRP and 2) had a control group that received the same SRP as the treatment group alone or with a placebo. We considered any type of probiotics with any type of administration method.	2010 to 2020	11	RCTs	Not reported	Descriptive analysis and meta-analysis	Primary outcomes: PPD, CAL, and BOP parameters Secondary outcomes: PI, GI or GBI, oral malodor parameters, microbiological effects, the progression and prognosis of disease, and the need for additional periodontal treatment	Favors probiotics
Liu et al. (2022)	To evaluate the effect of probiotics on gingival inflammation and oral microbiota in patients with plaque-induced gingivitis.	554 patients were included: 276 in the test group and 278 in the control group. Plaque-induced gingivitis patients with no history of periodontitis (periodontal pockets ≤3 mm, without clinical attachment loss)	The test group took oral probiotic lozenges or probiotic beverages, and the control group took oral placebo lozenges or placebo beverages.	2009 to 2020	11	RCTs	Not reported	Qualitative analysis and meta-analysis	GI, PI, BOP, GCF volume, the concentration of IL-1β in GCF, Aa count, Pg count, Pi count, and Fn count	Does not favor probiotics
Gao et al. (2020)	To evaluate the additional effect of probiotic LB in the nonsurgical management of peri-implant diseases (peri-implant mucositis and peri-implantitis).	Qualitative analysis: 285 participants with 296 implants. Meta-analysis: 233 patients with 244 implants, patients with peri-implant diseases who received nonsurgical treatment	Intervention, LB agent	2015 to 2019	7	RCTs	Spain, Japan, Italy, Sweden, and the United States	Qualitative synthesis and meta-analysis	Primary outcome: PPD Secondary outcome: BOP, PI, and microbiological parameters	Favors conventional approach
Priyanka et al. (2016)	Aim of this systematic review was to analyze the available scientific evidence on the effects of probiotics in prevention and treatment of periodontal diseases.	788 participants Anyone who received probiotics as a preventive or treatment agent for periodontal diseases (gingivitis or periodontitis)	Oral probiotic administration compared with placebo, no treatment, or another active intervention	2008 to 2015	15	RCTs	Not reported	Qualitative analysis	Outcome variables: PPD, CAL, PI, gingival inflammation, and BOP	Favors probiotics
Hardan et al. (2022)	The research question was: “Does the use of probiotics as adjuvant therapy for scaling and root debridement improve the clinical periodontal parameters?”	Total = 1,089 Patients with periodontal disease	Effect of the use of probiotics as adjuvants in the treatment of periodontal disease; included a control group where only scaling and root debridement was performed	2008 to 2022	25	RCTs	Not reported	Meta-analysis and subgroup analysis	PI, PPD, CAL, and BOP	Favors probiotic
Seminario-Amez et al. (2017)	To review the published literature with the purpose of knowing the importance of using probiotics as a preventive and therapeutic method for oral infectious diseases management.	1,291 patients of whom 380 were older than 18 y and 911 were minors	RCTs that assess the action of any probiotic strain in the treatment and/or prevention of an infectious oral disease, the action of any probiotic strain on counting the CFUs of oral pathogens, systematic reviews, and meta-analysis	2001 to 2016	15	RCTs	Not reported	Qualitative analysis	CFU counts of periodontal pathogens	Favors probiotics
Canut-Delgado et al. (2021)	Evaluating the effect of probiotics as an adjuvant to SRP, analyzing the effect of probiotics on the composition of the subgingival microbiota, and assessing possible short- or long-term side effects for the patient.	The number of patients included in the sample is between 20 and 51 and they are between 25 and 68 y old. (Table 3 describes *N* by each study, but not provided in online version of the paper.)	Probiotics as an adjuvant to SRP	Table 3 not provided in online version of the paper	10	RCTs	Table 3 not provided in online version of the paper	Qualitative analysis	PPD, PI, GI, CAL, BOP, microbiological effects, and immunological effects	Favors probiotics
Yanine et al. (2013)	Objective: This study was designed to determine the effects of probiotics in prevention and/or treatment of periodontal diseases.	188 participants Anyone who received probiotics as a preventive or treatment agent for periodontal diseases (gingivitis or periodontitis)	This study was designed to determine the effects of probiotics in prevention and/or treatment of periodontal diseases. Selecting of articles that satisfied the description of randomized clinical trials comparing the administration of probiotics versus placebo or another intervention to prevent or treat periodontal diseases in adult patients Oral probiotic administration compared with placebo, no treatment, or another active intervention	2008 to 2010	4	RCTs	Japan, India, and Germany	Due to the clinical heterogeneity of the studies, we considered that it was not appropriate to perform meta-analyses.	Primary outcome: PPD and CAL Secondary outcome: PI, GI, and BOP	Favors conventional approach
Donos et al. (2020)	This systematic review investigated the efficacy of host modulators combined with NSPT in reducing PPD in periodontitis patients.	Adults (≥18 y old), systemically healthy individuals diagnosed with periodontitis For probiotic outcome was 176 patients (5 RCTs)	Types of interventions (test group): studies evaluating the use of host modulators (modulators of inflammation, prebiotics, probiotics, antioxidant micronutrients) administered either topically or systemically in combination with NSPT	1993 to 2018; for probiotics, outcome from 2015 to 2018	58	RCTs	Not reported	Qualitative analysis and quantitative analysis	Primary outcome: reduction in PPD Secondary outcomes: 1) gain in CAL, 2) changes in bleeding indices, 3) changes in plaque indices, 4) radiographic bone defect changes (site level), 5) changes in GCF volume and markers, 6) patient-reported outcomes, including adverse events and adverse reactions as reported by the authors. For plaque and bleeding scores, all different indices reported by the authors were considered.	Favors placebo
Saïz et al. (2021)	The objective of this review was to assess the benefits of probiotics in oral health and disease, and in dental practice.	Total = 5,374 Type of participants: of any age (adults, children, the elderly), without gender restriction, healthy or not	Type of intervention: use of any probiotic (alone or in combination)	Not reported	91	RCTs	Not reported	Qualitative analysis	Primary outcomes: clinical, microbiological, immunological, and biochemical parameters Secondary outcomes: any adverse effects, rate of adherence, quality of life	Favors probiotic
Jayaram et al. (2016)	This review was performed to determine whether administration of probiotics produced a lasting clinical benefit in the treatment of periodontal disease.	Healthy volunteers or patients with periodontal disease (gingivitis, chronic periodontitis, or aggressive periodontitis)	Administration of probiotics in the treatment of periodontal disease	2008 to 2015	14	RCTs	Not reported	Qualitative analysis	Clinical benefits in the treatment of periodontal disease	Favors placebo
Martin-Cabezas et al. (2016)	“What is the short-term clinical influence of probiotic as an adjunctive therapy of SRP, in terms of PPD reduction and CAL gain, when compared with SRP alone or in combination with placebo in the treatment of CP in humans?”	130 participants Patients with CP	Intervention: probiotic as an adjunctive therapy of SRP; RCTs comparing SRP + probiotic versus SRP were included. The meta-analysis estimated PPD reduction (mean PPD; moderate and deep pockets), CAL gain, and reduction of percentage of sites with BOP expressed as the average difference between baseline and follow-up.	2010 to 2015	4	RCTs	Turkey and India	Descriptive analysis and meta-analysis	Primary outcome: PPD and CAL Secondary outcome: BOP, PI, GI, GBI, need for surgery, and risk of disease	Favors probiotic
Ikram et al. (2018)	The aim of the present study was to evaluate the efficacy of probiotics as an adjunct to SRP in the treatment of chronic periodontitis.	220 patients (individuals with CP)	Use of probiotics in adjunct to SRP	2010 to 2016	7	RCTs	Not reported	Qualitative analysis and quantitative analysis	Primary outcomes: changes in PPD reduction and CAL gain Secondary outcomes: PI, BOP, and GI	Favors probiotics
Sayardoust et al. (2022)	Primary aim: evaluate the potential microbiological effect of probiotics on the implant microbiota. Secondary aim: evaluate if probiotics have any effect as an adjunct to nonsurgical peri-implant treatment in reducing peri-implant mucositis and peri-implantitis clinical parameters—BOP, mGI, and PPD.	236 participants Human subjects with oral implants replacing missing teeth were included in the study. There was no preselected cohort based on a specific risk factor or studies evaluating an implant system or implant components.	To investigate the impact of probiotics, the test group is administered with probiotics and the control group with placebo. Probiotics may be added to the conventional treatment of mucositis and peri-implantitis.	2015 to 2020	7	RCTs	Not reported	Qualitative synthesis and meta-analysis	Primary outcomes: changes in microbiological composition (abundance of bacteria and/or diversity) Secondary outcomes: changes in the clinical peri-implant variables BOP, mGI, and PPD	Does not favor probiotics
Barootchi et al. (2020)	To assess the effectiveness of different nonsurgical protocols for the treatment of peri- implant mucositis.	*N* not reported for all studies (*n* for implants is presented). Patient diagnosed with peri-implant mucositis around implants supported restoration.	Effect of nonsurgical therapy alone in treating peri-implant mucositis Effect of the nonsurgical therapy with the adjunctive effect of chlorhexidine, glycine powder air-polishing, probiotic bacteria and photodynamic therapy	1997 to 2018	14	RCTs	Not reported	Qualitative analysis and meta-analysis	Primary outcomes: improvement of clinical parameters (PPD, BOP, PI, BI) after nonsurgical mechanical therapy Secondary outcomes: improvement of clinical outcomes after nonsurgical therapy alone versus additional therapies of mechanical debridement to treat peri-implant mucositis	Does not favor probiotics
Mishra et al. (2021)	To establish the significance of probiotic usage, both as a preventive as well as a therapeutic strategy for the management of periodontal disease. It also substantiates the existing studies of single/combined bacterial strain for exhibiting variable ecological impact on oral bacteria.	The number of participants in the included studies ranged between 28 and 60 and with an age range of 25 to 79 y. A total number of 529 participants enrolled in the studies but following dropouts, only 497 were analyzed.	RCTs using some form of probiotic therapy to treat periodontal disease, aiming to establish the significance of probiotic studies of single/combined bacterial strain for exhibiting variable ecological impact on oral bacteria. Intervention—receiving probiotics in addition to SRP as intervention.	2010 to 2019	14	RCTs	Not reported	Qualitative analysis and quantitative analysis	CAL and PD	Favors probiotic
Corbella et al. (2021)	The aim of this paper is to systematically review the literature on the efficacy of systemic HMs as adjuncts to NSPT in improving PPD reduction and CAL gain in healthy and systemically compromised patients.	2,431 participants Study population: adult (≥18 y old) patients affected by periodontitis, either systemically healthy or systemically compromised (e.g., with type 2 diabetes mellitus)	Test group—NSPT protocol (including mechanical treatment using manual curettes and/or ultrasonic devices without the use of antimicrobial agents) combined with the use of a systemic host modulator including but not limited to NSAIDs, bisphosphonates, unsaturated fatty acids, statins, sub-antimicrobial dose of doxycycline, probiotics, micronutrients, melatonin; control group—the same NSPT protocol alone or associated with a placebo	2004 to 2020	38	RCTs	Not disclosed for most studies	Qualitative analysis and quantitative analysis	Primary outcomes: reduction in PPD and/or CAL gain collected at patient level Secondary outcomes: changes in plaque scores, bleeding/inflammation scores, adverse events, and patient-reported outcome measures	Does not favor probiotic
Ho et al. (2020)	This systematic review aimed to evaluate the clinical, microbiological, and immunological outcomes of probiotics applied as an adjunct to NSPT with at least 3 mo of follow-up.	360 patients Adult patients who were diagnosed with periodontitis	Probiotics used as an adjunct in NSPT (scaling and root surface debridement + probiotics as test group)	2013 to 2018	10	RCTs	Not reported	Qualitative analysis and quantitative analysis	Primary outcome: CAL and PPD Secondary outcome: microbiological and immunological	Favors placebo
Matsubara et al. (2016)	Therefore, the aim of this systematic review was to explore the available clinical evidence on the efficacy of probiotic therapy in managing chronic periodontitis.	452 participants Patients affected by periodontitis	Intervention: oral administration of probiotic bacteria	2007 to 2016	12	RCTs	Chile, Belgium, Turkey, Sweden, Canada, Spain, India, and Japan	Qualitative synthesis	PPD, REC, GI, GBI, PI, BOP and CAL; qualitative and quantitative analysis of the burden of intraoral periodontal pathogens	Favors probiotic
Teughels et al. (2011)	This review was initiated to explore whether the use of probiotics can influence the periodontal microbiota and periodontal health.	3 animal and 11 in vivo human trials: 24 gnotobiotic rats; 48 beagle dogs Humans: 754 patients (with periodontal disease, but good general health)	The clinical effects of oral probiotics on periodontal health were reviewed systematically.	2003 to 2009	14	5 RCTs 2 open-label follow-up studies 3 nonrandomized clinical trials	Not reported	Qualitative analysis	Microbiological changes; changes in PI, GI, BOP, PPD, and CAL; inflammatory markers; other effects	Favors placebo
Akram et al. (2020)	Focused question—in subjects with gingivitis, what is the therapeutic efficacy of probiotics compared with placebo on gingival inflammatory parameters with only RCTs taken into consideration?	Probiotic group: 261 patients Placebo: 238 patients	Trials comparing the effectiveness of probiotics (in any form) with placebo	2006 to 2018	10	RCTs	Sweden, Spain, Turkey, Denmark, Italy, Germany, and Finland	Qualitative analysis and meta-analysis	Primary outcome: measure comprised the GI and/or BOP Secondary outcome: measures included PI. To address the aim of this study broadly, parameters such as GI, BOP, and PI were further reported.	Favors placebo
Gheisary et al. (2022)	The purpose of our SR and MA was to combine results from RCTs involving adults with periodontal diseases or healthy volunteers receiving probiotic supplementation to assess the effects on the clinical, microbiological, and immunological outcomes.	2,448 Adults 18 y of age or older, clinically diagnosed with periodontal disease or healthy adults (without periodontal disease)	Probiotic supplementation is the intervention group of the study.	2005 to 2020	64	RCTs	Turkey, Saudi Arabia, Iran, Italy, India, United Arab Emirates, Sweden, Pakistan, Spain, Brazil, Japan, Finland, Denmark, Belgium, Korea, Macedonia, Chile, Egypt, Hong Kong, Slovenia, Germany, and Indonesia	Meta-analysis and subgroup analysis	Clinical outcomes: PI, GI, PPD, CAL, BOP, REC, GCF volume Subgingival microbiological count: Pi, Fn, Tf, Pi, Aa, SM, and LB species GCF levels of immunological outcomes: matrix metalloproteinase 8, IL-6, IL-1β, IL-8, IL-10, and tumor necrosis factor α	Favors probiotic

Aa, *Aggregatibacter actinomycetemcomitans*; BI, bleeding index; BOP, bleeding on probing; CAL, clinical attachment level; CFU, colony-forming units; CP, chronic periodontitis; Fn, *Fusobacterium nucleatum;* GBI, gingival bleeding index; GCF, gingival crevicular fluid; GI, gingival index; HM, host modulator; IL, interleukin; LB, *Lactobacillus;* LR, *Lactobacillus reuteri*; MA, meta-analysis; mGI, modified gingival index; NSAID, nonsteroidal anti-inflammatory drug; NSPT, nonsurgical periodontal treatment; Pg, *Porphyromonas gingivalis*; Pi, *Prevotella intermedia*; PI, plaque index; PD, peri-implant diseases; PPD, periodontal probing depth/probing pocket depth; RCT, randomized controlled trial; REC, gingival recession; SD, subgingival debridement; SM, *Streptococcus mutans*; SR, systematic review; SRP, scaling and root planning; Tf, *Tannerella forsythia*. y, year.

Five SRs evaluated *Lactobacillus reuteri* individually (Martin-Cabezas et al. 2016; Silva et al. 2020; Song and Liu 2020; Arbildo-Vega et al. 2021; Sayardoust et al. 2022), and 4 SRs assessed a combination of *Lactobacillus reuteri* and other 2 subspecies of *Lactobacillus* (*brevis*, *planatarum*, *salivarus*, or *casei*) (Yanine et al. 2013; Ikram et al. 2018; Barootchi et al. 2020; Gao et al. 2020). Twenty SRs analyzed the effectiveness of a combination of different species and subspecies of probiotics, such as *Lactobacillus*, *Bifidobacterium*, *Streptococcus*, *Bacillus*, *Clostridium*, *Saccharomyces*, and *Pediococcus* (Teughels et al. 2011; Gruner et al. 2016; Jayaram et al. 2016; Matsubara et al. 2016; Priyanka et al. 2016; Seminario-Amez et al. 2017; Akram et al. 2020; Barboza et al. 2020; Donos et al. 2020; Ho et al. 2020; Vives-Soler and Chimenos-Kustner 2020; Abdulkareem et al. 2021; Corbella et al. 2021; Hu et al. 2021; Mishra et al. 2021; Saïz et al. 2021; Gheisary et al. 2022; Hardan et al. 2022; Liu et al. 2022; Ng et al. 2022). One SR did not report the probiotic species (Canut-Delgado et al. 2021).

### Risk of Bias within Included Studies

The Cohen’s κ interrater reliability for the 31 SRs subjected to the JBI Critical Appraisal tool was 0.95 (*P* = 0.018), considered an almost perfect agreement. After the overall appraisal, no SR was excluded. However, 6.4% of the studies (2) scored 49% or less, with eligibility criteria definition and search strategy as the most concerning items. See the detailed appraisal rating for randomized controlled trials (RCTs) included in the considered SRs in Appendix 5.

### Results of Individual Studies

Table 3 summarizes the results of individual SRs. The studies ranged in the period of administration of the probiotic therapy from a single dose (Teughels et al. 2011) to 6 months (Silva et al. 2020). The follow-up period ranged from 1 day (Teughels et al. 2011) to 9 years (Gruner et al. 2016). The included SRs reported no clinical data about bone loss around teeth or implants, implant survival rate, and tooth loss outcomes. Sixteen SRs advocated the clinically relevant effect of probiotic therapy as an adjuvant to SRP for treating periodontal disease (Martin-Cabezas et al. 2016; Matsubara et al. 2016; Priyanka et al. 2016; Seminario-Amez et al. 2017; Ikram et al. 2018; Barboza et al. 2020; Song and Liu 2020; Vives-Soler and Chimenos-Kustner 2020; Arbildo-Vega et al. 2021; Canut-Delgado et al. 2021; Hu et al. 2021; Mishra et al. 2021; Saïz et al. 2021; Gheisary et al. 2022; Hardan et al. 2022; Ng et al. 2022). Twelve SRs did have low risk of bias (Martin-Cabezas et al. 2016; Matsubara et al. 2016; Ikram et al. 2018; Barboza et al. 2020; Song and Liu 2020; Arbildo-Vega et al. 2021; Hu et al. 2021; Mishra et al. 2021; Gheisary et al. 2022; Hardan et al. 2022; Ng et al. 2022), 4 did have moderate risk of bias (Priyanka et al. 2016; Vives-Soler and Chimenos-Kustner 2020; Canut-Delgado et al. 2021; Saïz et al. 2021), and 1 had a high risk of bias (Seminario-Amez et al. 2017). The probiotic therapy administration period in the included studies ranged from a single dose (Hu et al. 2021) to 7 months (Seminario-Amez et al. 2017). The follow-up period ranged from 4 days (Barboza et al. 2020) to 4 years (Seminario-Amez et al. 2017).

**Table 3.** Results of Individual Studies.

Author (Year)	Probiotic	Form of Administration	Time of Administration	Follow-up Period	Substrate	Systemic Disease	Critical Appraisal	Heterogeneity	Results	Effect Sizes (95% CI)	Confidence on Evidence
Silva et al. (2020)	*Lactobacillus reuteri*	Oil	3 wk to 6 mo	1 to 6 mo	Implant	Considered in the exclusion criteria	Cochrane Collaboration risk of bias tool CONSORT statement	Not applicable Due to the clinical heterogeneity of the studies, we considered meta-analysis to be inappropriate as trials that assessed the same outcomes used different follow-up times and/or biases were observed. As such, results were not considered to be comparable.	Five randomized clinical trials were analyzed in the final review process. For the primary outcomes (PPD and BOP) and for the secondary outcomes (plaque index, gingival index, gingival crevicular fluid and microbiological tests), no significant clinical effects of probiotics were observed.	NR	NR
Abdulkareem et al. (2021)	*Bifidobacterium animalis subsp. Lactis*, *Lactobacillus reuteri*, *Streptococcus faecalis*, *Bacillus mesentericus*, *Lactobacillus sporogenes*, *Lactobacillus brevis*, *Lactobacillus plantarum*, *Lactobacillus rhamnosus*	Lozenge, drops, and sachet	3 wk to 3 mo	1 mo to 5 ys	Tooth	Not considered as a criterion	NR	Not applicable	Evidence from in vitro, observational, and clinical trial studies suggests efficacy in the use of adjunctive antimicrobials in patients with grade C periodontitis of young age or where the associated risk factors are inconsistent with the amount of bone loss present. Meanwhile, alternative approaches such as photodynamic therapy, bacteriophage therapy, and probiotics showed limited supportive evidence, and more studies are warranted to validate their efficiency.	NR	NR
Vives-Soler and Chimenos-Kustner (2020)	*Streptococcus oralis (KJ3)*, *S. uberis (KJ2)*, *S. rattus (JH145)*, *Lactobacillus reuteri*, *L. reuteri + L. salivarius*, *L. rhamnosus SP1*, *L. plantarum HK*	Lozenge, mouthwash, capsule, topical application	2 wk to 3 mo	12 wk to 1 y	Tooth and Implant	Not considered as a criterion	Cochrane Collaboration risk of bias tool	Not applicable	Nine trials were included. A narrative data synthesis did not result in any major improvement of overall pocket probing depth, but moderate pockets from 4 to 6 mm showed larger reductions in study groups, which could decrease the need for surgery. Sites with bleeding on probing and presence of plaque decreased after treatment. For peri-implant mucositis, there was a small tendency to better results in the study group. With the available data, it is concluded that probiotics may provide an additional benefit to manual debridement in chronic periodontitis. More studies are required on dose, route of administration, and strains of probiotics used.	NR	NR
Arbildo-Vega et al. (2021)	*Lactobacillus reuteri DSM 17938* and *LR ATCC PTA 5289*	Pills and drops	3 wk to 6 mo	3 to 6 mo	Implant	Not considered as a criterion	Cochrane Collaboration risk of bias tool	*I*^2^ statistics and χ^2^ test A random effects model was used for the meta-analysis in the treatment of peri-implant mucositis, due to the heterogeneity that existed between each of the studies.	The search strategy resulted in 6 articles, of which 4 investigated peri-implantitis and 3 peri-implant mucositis. All studies reported that there was a difference in the depth of the probing in the treatment of PD, in favor of the group using LR, though not always achieving significance.	Peri-implant mucositis: Total = 0.12 (0.01/0.22),*I*^2^ = 78% PPD = 0.27 (–0.19/0.74),*I*^2^ = 83% Peri-implantitis: Total = 0.08 (0.01/0.16),*I*^2^ = 0% PPD = 0.26 (–0.01/0.52),*I*^2^ = 6%	NR
Song and Liu (2020)	*Lactobacillus reuteri*	Lozenges, tablet, and sachet (powder)	3 wk to 3 mo	1 month to 1 y	Tooth	Considered in the exclusion criteria	Cochrane Collaboration risk of bias tool	Not reported, but calculated in the forest plot, but does not mention how to deal with heterogeneity	The meta-analysis plots were used to assess all the clinical outcomes. The mean difference of reduction in PPD at 21 d (MD, 0.61) and 3 mo (MD, 0.40), and CAL gain at 3 mo (MD, 0.30) showed favorable response in the sites treated with probiotics containing *Lactobacillus reuteri* in addition to SRP. The meta-plots for major peridonto-pathogens constructed at 21-d follow-up showed short-term effective reduction.	PPD (6 mo) = 0.56 (–0.06/1.18),*I*^2^ = 99% CAL (6 mo) = 0.08 (–0.12/0.28),*I*^2^ = 78% Micro (6 mo) = 0.84 (0.69/0.99),*I*^2^ = 82%	NR
Hu et al. (2021)	*Bifidobacterium animalis subsp. Lactis; Lactobacillus reuteri; Streptococcus oralis*, *uberis*, and *rattus; Lactobacillus rhamnosus; Streptococcus faecalis; Clostridium butyricum; Bacillus mesentericus; Lactobacillus sporogenes; Lactobacillus casei shirota; Lactobacillus salivarius; Saccharomyces boulardii; Lactobacillus plantarum; Lactobacillus brevis; Lactobacillus plantarum; Streptococcus salivarius; Lactobacillus paracasei*	Lozenges, tablet, drink, mouthwash, gel, and sachet	Once to 4 mo	14 d to 12 mo	Tooth	Not considered as a criterion	Cochrane Collaboration risk of bias tool	The statistical heterogeneity across the included studies was assessed by using the Standard χ^2^ tests and *I*^2^ statistics. There was a significant heterogeneity in clinical outcome at 3 mo.	Twenty-four randomized controlled trials (RCT) were included in the meta- analysis. The pooled results showed CAL gain (WMD: 0.20; 95% CI, 0.09 to 0.31), PPD reduction (WMD: −0.31; 95% CI, −0.52 to −0.10), and BOP reduction (WMD: −2.98; 95% CI, –4.70 to −1.26) in the SRP+ probiotics group were significantly improved compared to control group at 3-mo follow-up, but no significant difference was observed at 6 mo. In addition, the probiotics administration could improve plaque index (WMD: −0.30; 95% CI, −0.59 to −0.05) and gingival index (WMD: −0.46; 95% CI, −0.71 to −0.21) at short term.	PPD (3 mo) = −0.31 (–0.52/–0.10),*I*^2^ = 95.3% CAL (3 mo) = 0.20 (0.09/0.31),*I*^2^ = 70.1% BOP (3 mo) = −2.98 (–4.70/–1.26),*I*^2^ = 2.6%	NR
Gruner et al. (2016)	*Lactobacilli*, *Bifidobacteria*, *Streptococci*, or *Bacilli*	Milk, milk products (curd, ice cream, cheese, yogurts), tablets, lozenges, candies/gum, nonmilk drinks or liquids, powders, straws, and cereals	14 d to 12 wk	2 d to 9 y	Tooth	Not considered as a criterion	Cochrane Collaboration risk of bias tool	Cochran’s *Q* and *I*^2^ statistics We found a great heterogeneity between trials.	50 studies (3,247 participants) were included. Studies were mainly performed in children and used lactobacilli (45); bifidobacteria (12) or other genus (3). Probiotics significantly increased the chance of reducing SM (OR: 2.20; 95% CI: 1.23 to 3.92) or LB (OR: 2.84; 1.34 to 6.03) <104 CFU/mL. Such reduction was confirmed for SM counts (standardized mean differences: −1.18; 95 CI, −1.64 to −0.72), but not LB (SMD: 0.33; 0.15 to 0.52). For periodontal pathogens, no significant difference was found. Probiotics significantly reduced bleeding on probing (SMD: −1.15; −1.68 to −0.62) and gingival index (SMD: −0.86; −1.52 to −0.20), but not plaque index (SMD: 0.51; −1.10 to 0.07). Caries incidence was not significantly reduced (OR: 0.60; 0.35 to 1.04), and neither was caries experience (SMD: −0.26; −0.55 to 0.03) or CAL (SMD: −0.46; −0.84 to 0.08). In contrast, probing pocket depths (SMD: −0.86; −1.55 to −0.17) were significantly reduced. Data were quantitatively insufficient for conclusive findings, and risk of bias was high.	BOP = −1.15 (–1.68 to −0.62),*I*^2^ = 47% Micro Aa = −0.33 (–1.32 to 0.66),*I*^2^ = 65% Pg = −0.29 (–0.77 to 0.19),*I*^2^ = 0% Pi = −0.48 (–1.49 to 0.53),*I*^2^ = 76%	GRADE assessment
Barboza et al. (2020)	*Lactobacillus casei Shirota; Lactobacillus reuteri; Lactobacillus brevis CD2; Bifidobacterium animalis subsp.*	Lozenges, milk drink (yogurt), and dairy drink	2 to 8 wk	4 d to 8 wk	Tooth	Not considered as a criterion	Cochrane Collaboration risk of bias tool	Not applicable. No meta-analysis could be conducted due to the heterogeneity of the selected studies.	A total of 5 articles were included in the qualitative synthesis. No meta-analysis could be conducted due to the heterogeneity of the selected studies. The use of probiotics showed a slight improvement in clinical parameters. Changes in gingival crevicular fluid volume were lower in the presence of the probiotic than in the placebo group. All the studies showed that the immediate, positive effects of probiotics during the period of discontinued mechanical oral hygiene were due to the modulation of the host response, not the antiplaque effect.	NR	NR
Ng et al. (2022)	*Lactobacillus reuteri*, *Lactobacillus salivarius*, *Lactobacillus acidophilus*, *Lactobacillus rhamnosus SP1*, *Bifidobacterium lactis HN019*, *Streptococcus oralis KJ3*, *Streptococcus uberis KJ2*, *Streptococcus rattus JH145*	Lozenges	28 d to 3 mo	3 mo and 1 y	Tooth	Considered in the exclusion criteria	Cochrane Collaboration risk of bias tool	Cochran’s *Q* test and *I*^2^ statistic PPD *I*^2^ = 86%	Ten studies were selected from 818 records. Meta-analysis showed that adjunctive probiotics had no additional benefit for percentage change of the total number of deeper sites (5 mm, 6 mm, 7 mm) before and after therapy. No significant difference was observed for mean probing pocket depth reduction at 3 and 6 mo. Statistically significant beneficial odds ratios for need for additional therapy (OR, 0.19; 95% CI, 0.07–0.56) and risk of disease progression (OR, 0.32; 95% CI, 0.14–0.73) were observed with probiotic administration. Immunological rather than microbiological outcomes correlated more consistently with clinical findings. No adverse events were reported.	PPD (12 mo) = −0.83 (–1.48 to −0.18),*I*^2^ = 93%	NR
Gou et al. (2020)	*Lactobacillus reuteri; Lactobacillus rhamnosus SP1; Lactobacillus plantarum; Lactobacillus salivarius+ Lactobacillus reuteri; L. salivarius NK02; Streptococcus oralis; KJ3+ Streptococcus uberis; KJ2+ Streptococcus rattus JH145; Bifidobacterium animalis subsp. Lactis (B. lactis); HN019 probiotic*	Lozenges, sachet, and mouthwash	14 d to 3 mo	42 d to 12 mo	Tooth	Considered in the exclusion criteria	Cochrane Collaboration risk of bias tool	Not reported, but calculated in the forest plot, but does not mention how to deal with heterogeneity	After screening, 11 publications were eligible for the systematic review and 10 were evaluated in the meta-analysis. Results demonstrated statistically significant more overall PPD reduction at 1 mo (0.48 mm, *P* = 0.005), overall CAL gain at 1 (0.35 mm, *P* = 0.004) and 3 mo (0.13 mm, *P* = 0.04), and BOP percentage reduction (10.38, *P* = 0.001) at short term and 6 mo (7.57, *P* < 0.00001) favoring SRP + probiotics treatment. Moreover, significantly more reduction of PPD for moderate (0.19 mm, *P* < 0.00001) and deep pockets (0.58 mm, *P* < 0.00001) and gain of CAL for moderate pockets (0.20 mm, *P* = 0.0001) were observed at 3 mo favoring adjunctive efficacy of probiotics. However, there were not a significant difference of overall PPD reduction at 3 (0.14 mm, *P* = 0.07) and 6 mo (0.2 mm, *P* = 0.26) and overall CAL gain at 6 mo (0.19 mm, *P* = 0.53) between 2 groups.	PPD (6 mo) = −0.20 (–0.54 to 0.14), *I*^2^ = 91% CAL (6 mo) = −0.19 (–0.75 to 0.38), *I*^2^ = 97% BOP (6 mo) = −7.57 (–8.64 to −6.50),*I*^2^ = 47%	NR
Liu et al. (2022)	*LGG*, *BB-12*, *Bifidobacterium animalis subsp. Lactis*, *L. reuteri*, *Bacillus coagulans*, *Lactobacillus rhamnosus*, *Lactobacillus curvatus*, *L. brevis*, *L. plantarum*, *L. brevis*, *P. acidilactici*, *Lactobacillus casei strain Shirota*	Lozenge, yogurt, and tablet	14 d to 3 mo	14 to 56 d	Tooth	Considered in the exclusion criteria	Cochrane Collaboration risk of bias tool	*I*^2^ test	All comparisons displayed that oral probiotic had no significant improvement in the GI, PI, and BOP of patients with plaque-induced gingivitis. In terms of microecology, no significant difference in the volumes of GCF, the concentration of IL-1β, and the counts of Aa, Pg, Pi, and Fn were found between the probiotic group and the placebo group.	BOP = 0.07 (−0.04 to 0.18), *I*^2^ = 0% Micro Aa = 0.01 (−0.55 to 0.58),*I*^2^ = 0% Pg = −0.29 (−0.75 to 0.17),*I*^2^ = 43% Pi = −0.15 (−0.80 to 0.50),*I*^2^ = 0% Fn = −0.04 (−0.37 to 0.28), *I*^2^ = ?	NR
Gao et al. (2020)	*Lactobacillus reuteri*, *Lactobacillus brevis*, and *Lactobacillus plantarum*	NR	14 d to 6 mo	1 to 6 mo	Implant	Not considered as a criterion	Cochrane Collaboration’s risk of bias tool	Cochrane *Q* test and *I*^2^ Obvious heterogeneity was detected across studies. Immediately (*I*^2^ = 0%); 2 mo (*I*^2^ = 98%)	Seven randomized controlled trials with 296 implants were included in this meta-analysis. The mean difference of probing pocket depth (PPD) was –0.05 (95% CI: –0.28 to 0.18; P = .67) immediately after treatment termination and –0.17 (95% CI: –1.01 to 0.67, P = .69) at least 2 mo after treatment termination. There was a slight reduction of PPD after treatment termination. Compared with placebo, *Lactobacillus* provided limited benefits in peri-implant mucositis. There were no significant differences in the secondary outcomes of bleeding on probing plaque index (*P* > .05). In a narrative synthesis of peri-implantitis, the effect of *Lactobacillus* on PPD and bleeding on probing remained controversial.	PPD (2 mo) = −0.17 (–1.01 to 0.67), *I*^2^ = 98%	GRADE assessment
Priyanka et al. (2016)	*Lactobacillus salivarius WB21*, *Lactobacillus casei*, *Lactobacillus reuteri*, *Streptococcus salivarius*, *Lactobacllus sporogens*, *Streptococcus faecalis*, *Clostridium butyrium*, *Bacillus mesentricus*	Tablet, milk, chewing gum, lozenges	14 d to 3 mo	42 d to 3 mo	Tooth	Not considered as a criterion	Cochrane Collaboration’s risk of bias tool	Not applicable	The initial search resulted in 73 articles; however, 45 of these articles were excluded after reviewing the abstracts because they did not have the proper clinical trial design or because they were duplicates. After analyzing the full text from 27 clinical trials, 12 were excluded because they did not fulfill all the selection criteria. Our final review included 15 articles. Included outcome measures were probing pocket depth, bleeding on probing, clinical attachment loss, plaque index, and gingival inflammation. Included studies were subjected to critical analysis following the Cochrane Collaboration tool for evaluating the risk of bias.	NR	NR
Hardan et al. (2022)	*Lactobacillus brevis*, *Lactobacillus plantarum*, *Lactobacillus reuteri*, *Bifidobacterium animalis*, *Weissella cibaria*, *Lactobacillus salivarius*, *Lactobacillus rhamnosus*, *Lactobacillus rhamnosus*, *Lactobacillus brevis*, *Lactobacillus plantarum*, *Lactobacillus reuteri*, and *Bifidobacterium*	NR	6 d to 6 mo	4 to 24 wk	Tooth	Not considered as a criterion	Cochrane Collaboration’s risk of bias tool	Cochran *Q* test and the inconsistency *I*^2^ test	For the index plaque, the use of probiotics did not improve this clinical parameter (*P* = 0.16). On the other hand, for the periodontal pocket depth, the clinical attachment loss, the bleeding on probing, and the use of probiotics as adjuvant therapy resulted in an improvement of these parameters, since the control group achieved statistically higher values of this parameter (*P* < 0.001, *P* < 0.001, and *P* = 0.005, respectively).	PPD (12 mo) = −0.44 (0.22–0.66),*I*^2^ = 70% CAL loss (12 mo) = 0.44 (0.30–0.58),*I*^2^ = 29% BOP (12 mo) = 0.46 (0.14 to −0.78),*I*^2^ = 86%	NR
Seminario-Amez et al. (2017)	*S. oralis*, *S. uber*, *S. sattus*, *L. reuteri*, *L. paracasei*, *B. lactis*, *L. acidophilus*, *L. brevis*, *B. lactis (Bb-12)*, *L. salivarius*, *L. rhamnosus*, *Bifidobacteria*, *L. rhamnosus*, *L. rhamnosus*, *B. longum*, *Saccharomyces cereviasae*, *Bacilus coagulans*, *L. sporogenes*	Tablets, ice cream, milk, sachet, powder	7 d to 23 mo	15 d to 4 y	Tooth	Not considered as a criterion	Jadad scale	Not applicable	Probiotics are a kind of bacteriotherapy that, according to the literature reviewed, provides a decrease in CFU counts of cariogenic pathogens (*S. mutans*). Regarding periodontal disease, the studies included in this review reported a clinical improvement of bleeding on probing, probing depth, and gingival index but no significant difference in CFU counts of periodontal pathogens. Anyway, it is important to highlight that these diseases have a multifactorial etiology, which means that reducing the CFU counts does not ensure their absolute control. RCTs with homogeneous methodologies and long-term follow-up periods are needed to confirm their contribution in the management of these diseases and their influence in their prevalence. Furthermore, the recognition of specific strains with probiotic activity for each infectious oral disease is required to determine exact dose, treatment time, and ideal vehicles.	NR	NR
Canut-Delgado et al. (2021)	NR	Lozenges, sachet	2 wk to 3 month	1 to 12 mo	Tooth	Considered in the exclusion criteria	Cochrane Collaboration’s risk of bias tool	Not applicable	In 7 trials, the clinical parameters evaluated were significantly improved in the test group compared with placebo. However, in 3 studies, no significant differences were reported between the 2 groups in the clinical parameters evaluated, but in 1 there was a significant improvement in PI and GI (Table 3). On the other hand, a representative reduction of the main periodontal pathogens was obtained in 4 clinical trials, and in 2 studies, there was a significant reduction in proinflammatory cytokines.	NR	NR
Yanine et al. (2013)	*Lactobacillus salivarius WB21*, *Lactobacillus reuteri*, and *Lactobacillus casei*	Tablet, gum, and milk	3 wk to 30 d	42 d to 8 wk	Tooth	Considered in the exclusion criteria	Cochrane Collaboration’s risk of bias tool	We assessed clinical heterogeneity based on the setting, patients, intervention, and outcome measurement characteristics. We used the risk of bias tool to evaluate methodological heterogeneity. We planned to assess statistical heterogeneity using the χ^2^ test and the *I*^2^ statistic. Due to the clinical heterogeneity of the studies, we considered that it was not appropriate to perform meta-analyses.	Four randomized clinical trials were analyzed in the final review process. For the primary outcome, probing pocket depth, there would be no clinical beneficial effect of probiotics. For secondary outcomes, probiotics have shown small benefits on plaque index and gingival inflammation.	NR	NR
Donos et al. (2020)	*L. reuteri*, *S. oralis KJ3*, *S. uberis*, *KJ2*, and *S. rattus JH145/da*, *rhamnosus*	Lozenges, tablets, and sachets	3 wk to 3 mo	6 to 12 mo	Tooth	Not considered as a criterion	Cochrane Collaboration’s risk of bias tool	Cochran’s test and *I*^2^ statistics	Probiotics chapter—meta-analysis suggested that, compared with placebo, treatment with probiotics resulted in a benefit in PPD reduction of 0.38 mm (95% CI, −0.14 mm to 0.90 mm). The mean prediction interval ranged from −1.61 to 2.37 mm. Funnel plot and Egger’s test (*P* = 0.15 for PPD) did not show evidence for small-study effects.	PPD = 0.38 (−0.14 to 0.90), *I*^2^ = ?	NR
Saïz et al. (2021)	*Bifidobacterium animalis subsp. Lactis*, *Bifidobacterium longum*, *Lactobacillus acidophilus*, *Bifidobacterium bifidum*, *Bifidobacterium lactis* and *acid lactic bacillus*, *Lactobacillus sporogenes*, *Streptococcus faecalis PC*, *Clostridium butyrium TO-A*, *Bacillus mesentericus*, *Lactobacillus rhamnosus*, *Lactobacillus curvatus*, *Lactobacillus brevis*, *Lactobacillus rhamnosus*, *Bifidobacterium lactis*, *Lactobacillus plantarum*, *Saccharomyces boulardii*, *Streptococcus oralis KJ3*, *Streptococcus uberis KJ2*, *Streptococcus rattus JH14*, *Streptococcus faecalis*	NR	Once to 3 mo	1 wk to 4 mo	Tooth and implant	Not considered as a criterion	NR	Not applicable	Despite major inconsistencies between clinical trials, probiotics have been found to contribute to reduce *S. mutans* counts (*L. paracasei SD1*), reduce probing depth in chronic periodontitis (*B. animalis subsp. lactis DN-173010* with *L. reuteri*), reduce levels of volatile sulfur compounds and halitosis (*L. salivarius WB21*), treat oral mucositis, and improve the quality of life of patients undergoing cancer chemo-radiotherapy (*L. brevis CD2*). Combinations of probiotic bacteria tend to lead to higher clinical efficacy than any individual probiotic agent.	NR	NR
Jayaram et al. (2016)	*Lactobacillus rhamnosus*, *Lactobacillus brevis*, *L. salivarius*, *B. subtilis*, *L. reuteri*, *S. oralis KJ3sm*, *S. uberis*, *S. rattus*	Tablet, lozenges, chewing gum, and chewable tablet	2 wk to 3 mo	14 d to 1 y	Tooth	Not considered as a criterion	CONSORT checklist	Not applicable	A total of 13 papers that addressed the question of the use of probiotics in the treatment of periodontal disease were retrieved. Most of the studies reviewed showed only a short-term benefit with regards to reduction in gingival inflammation and probing depth reduction. Lasting clinical benefits were not seen in any of the studies. At least 4 different combinations and strains of probiotics have been used in the studies.	NR	NR
Martin-Cabezas et al. (2016)	*Lactobacillus reuteri*	Lozenges	3 to 12 wk	42 d to 12 mo	Tooth	Considered in the exclusion criteria	Cochrane Collaboration’s risk of bias tool	Interstudy heterogeneity appeared significant regarding overall: PPD reduction (94%), CAL gain (67%), and BOP reduction (97%).	Independent screening resulted in 4 eligible publications for the systematic review and 3 were included in the meta-analysis. Meta-analysis showed a statistically significant CAL gain (0.42 mm, *P* = 0.002) and BOP reduction (14.66, *P* = 0.003) for SRP + probiotic treatment versus SRP at short term. Only a tendency (*P* = 0.06) has been observed in terms of overall PPD reduction, whereas results were significant when stratified for moderate (0.18, *P* = 0.001) and deep pockets (0.67, *P* < 0.001).	PPD (3 mo) = −0.46 (–0.95 to −0.02),*I*^2^ = 94% CAL (3 mo) = −0.42 (–0.68 to −0.16),*I*^2^ = 67% BOP (3 mo) = −14.66 (–24.49 to −4.83),*I*^2^ = 97%	NR
Ikram et al. (2018)	*Lactobacillus reuteri*, *Lactobacillus reuteri + Lactobacillus salivarius*	Tablet, mouthwash, oral lozenges	2 to 12 wk	3 to 52 wk	Tooth	Not considered as a criterion	Cochrane Collaboration’s risk of bias tool	*I*^2^ and χ^2^ statistics PD: *I*^2^ = 82.69% CAL: *I*^2^ = 89.61% Significant heterogeneity was observed for PPD reduction and CAL gain	Seven clinical studies were included. Four studies showed additional benefits in reducing PPD and gaining CAL, whereas 3 studies showed comparable clinical periodontal outcomes between probiotics and SRP/placebo. Significant heterogeneity was observed for PPD reduction and CAL gain. The overall mean difference for CAL gain between probiotics and placebo/SRP was significant (WMD = 1.41; 95% CI, 0.15–2.67; *P* = 0.028) at follow-up.	PPD = 0.66 (–0.36 to 1.69), *I*^2^ = 82.69% CAL = 1.41 (–0.15 to 2.67), *I*^2^ = 89.61%	NR
Shariel Sayardoust et al. (2022)	*Lactobacillus reuteri*	Tablet	30 d to 6 mo	28 d to 6 mo	Implant	Not considered as a criterion	Cochrane Collaboration’s risk of bias tool	Not measure, but it is discussed; authors used REM in MA.	The data synthesis showed that probiotics had no detectable effect on the implant microflora, and in the following data synthesis, no clinical peri-implantitis variable showed a significantly beneficial effect from probiotics in the test group compared to the control group.	PPD = −0.36 (−0.85 to 0.13), *I*^2^ = ?	NR
Barootchi et al. (2020)	*Lactobacillus plantarum*, *Lactobacillus brevis*, and *Lactobacillus reuteri*	Tablets	14 d to 3 mo	6 wk to 3 mo	Implant	Not considered as a criterion	Cochrane Collaboration’s risk of bias tool	χ^2^ and the *I*^2^ statistics test Considerable heterogenicity and the limited comparable articles in the meta-analysis restricted the power of the analysis and hence the reliability of our results.	Fourteen studies were included in the systematic review and 3 in the meta-analysis. None of the selected studies reported a complete resolution of the peri-implant mucositis lesions. A nonsurgical therapy alone showed an average reduction of 0.57 mm (95% CI, 0.30–0.83) in PPD, 2.41% (95% CI, 12.74–32.08) in BOP, 17.28% (95% CI, 3.99–30.58) in the PI, and 13.41% (95% CI, 3.50–23.31) in the BI. The meta-analysis failed to demonstrate significant improvements with the adjunct use of chlorhexidine disinfectant to nonsurgical mechanical debridement for PPD reduction (–0.07 mm; 95% CI, −0.33 to 1.15; *P* = 0.62) and relative attachment level gain (–0.13 mm; 95% CI, −0.6 to 0.35; *P* = 0.6).	NR	NR
Mishra et al. (2021)	*Lactobacillus reuteri*, *Lactobacillus brevis CD2*, *Bifidobacterium lactis*, *Lactobacillus rhamnosus*, *Lactobacillus salivarius*, *Lactobacillus acidophilous*	Lozenges, tablets, sachets (powder), mouthwash (plus subgingival irrigation) and packet (powder)	14 d to 3 mo	42 d to 12 mo	Tooth	Not considered as a criterion	Cochrane Collaboration’s risk of bias tool	Not applicable	Fourteen clinical studies were included and demonstrated efficacy in reducing periodontal PPD and gaining CAL, between probiotics and SRP/placebo. Adjunctive probiotic therapy in addition to SRP leads to decrease in probing depth and clinical attachment gain in chronic periodontitis patients. However, further high-quality randomized clinical trials with microbiological outcomes are required to fortify the conclusion.	NR	NR
Corbella et al. (2021)	*L. reuteri*, *S. oralis KJ3*, *S. uberis KJ2*, *S. rattus*, *L. rhamnosus SP1*, *B. lactis HN019*, *L. reuteri*, *L. acidophilus*	Tablet	21 d to 6 mo	3 to 12 mo	Tooth	Not considered as a criterion	Cochrane Collaboration’s risk of bias tool	*I*^2^ statistics and Conrad’s test In the meta-analysis, effect size was computed through the weighted mean method and results were combined using DerSimonian and Laird’s random-effect model, assuming heterogeneity among studies.	38 articles were included in the qualitative assessment and 27 of them were included in the meta-analysis. There is low/very low evidence that the adjunctive use of sub-antimicrobial dose of doxycycline, melatonin, and the combination of omega-3 and low-dose aspirin (in type 2 diabetic patients) to NSPT would improve PD and/or CAL. Conflicting evidence is available on the efficacy of probiotics. Future studies controlling for confounding factors, using composite outcomes to define the end point of therapy and considering not only the patient- but also as the site-specific effect of systemic HMs, are warranted. The dosage, posology, and long-term effect of HMs still need to be clarified, also in association with the presence of systemic conditions potentially affecting the response to HM administration.	PPD (12 mo) = 0.84 (0.22–1.46), *I*^2^ = ? CAL (12 mo) = 0.70 (0.36–1.04), *I*^2^ = ? BOP (12 mo) = 7.41 (2.34–12.49), *I*^2^ = ?	GRADE assessment
Ho et al. (2020)	*L. reuteri*, *Streptococcus oralis KJ3*, *Streptococcus uberis KJ2*, *Streptococcus ratus*, *L. salivarius*, *Lactobacillus rhamnosus*, *Streptococcus faecalis*, *Clostridium butyricum*, *Bacillus mesentericus*, *Lactobacillus sporogenes*, *L. paracasei*	Lozenge, tablet, mouth rinse, and sachet	3 wk to 4 mo	3 mo to 1 y	Tooth	Not considered as a criterion	Cochrane Collaboration’s risk of bias tool	*I*^2^ statistics and χ^2^ test High heterogeneity among the included studies	Ten randomized controlled trials were included, and high heterogeneity in methods was noted. Meta-analysis revealed CAL gain, and PPD reduction in the probiotics group was significant at 3 mo and 12 mo, but no significant difference was noted at 6 mo and 9 mo. There was no significant difference in periodontal pathogen levels between groups at 3 mo. Immunological data were not sufficient for quantitative analysis. Ancillary sensitivity analysis indicated a subset of studies with severe mean baseline PPD (≥5 mm) at baseline showed significant and more CAL gain and PPD reduction at 3 mo, with probiotics administration of 2 to 4 wk.	PPD (12 mo) = −1.16 (–1.32 to −0.99),*I*^2^ = 0% CAL (12 mo) = 0.90 (0.79–1.02),*I*^2^ = 0% Micro Pg = 0.15 (–0.31 to 0.60), *I*^2^ = 93.8% Tf = 0.27 (–0.24 to 0.77),*I*^2^ = 0% Pi = −0.00 (–0.75 to 0.74), *I*^2^ = 51.2% Fn = −0.06 (–0.41 to 0.30), *I*^2^ = 27.1%	NR
Matsubara et al. (2016)	*Lactobacillus reuteri*, *Lactobacillus salivarius*, *Lactobacillus brevis*, *Lactobacillus rhamnosus*, *Streptococcus oralis*, *S. uberis*, and *S. rattus* species	Lozenges, tablets, sachet, or as suspensions in soybean oil	4 d to 3 mo	15 d to 12 mo	Tooth	Not considered as a criterion	Cochrane Collaboration’s risk of bias tool	Not applicable	All trials that used *Lactobacillus* species as probiotics yielded favorable clinical outcomes in terms of a reduction of the conventional disease indices such as PPD, BOP, GI, and PI, with probiotic administration and concomitant scaling and root planning (conventional mechanical treatment). One RCT testing *Streptococcus* spp. as a probiotic showed no significant difference between the probiotic and the placebo groups regarding PPD, BOP, GI, and CAL, although *P. intermedia* counts were reduced in the probiotic group after 12 wk of treatment. Oral administration of lactobacilli significantly reduced (*P* < 0.05) the oral burden of periodontal pathogens, such as Aa, Pg, and Pi. The maintenance of this beneficial effect was dependent on continuous probiotic consumption. No adverse effects occurred. Positive compliance to the treatment in 7 studies.	NR	NR
Teughels et al. (2011)	*S. sanguinis KJ3sm*, *S. salivarius*, *S. mitis*, *L. reuteri*, *W. cibaria CMS1*, *Lactobacillus casei*, *S. oralis KJ3sm*, *S. uberis KJ2sm*, *S. rattus JH145*, and *L. brevis (CD2)*	NR	Single application to 12 wk	1 day to 3 mo	Tooth	Not considered as a criterion	NR	Not applicable	Microbiological changes—controversial results, 1 study concluded that periodontopathogenic bacteria in the probiotic group was significantly decreased in subgingival plaque after 4 wk of probiotic usage and tended to be lower after 8 wk when compared with the placebo group. Other authors considered that probiotic did not influence the number of bacteria counting. But also depends on the type of bacteria analyzed. BOP—All 3 human studies that report on bleeding upon probing show significant decreases when compared with baseline values. PPD and CAL—Of the 2 human studies that reported on changes in PPD, only the study by Shimauchi et al. (2008) could detect statistically significant greater improvements in PPD for the probiotic group, but only for current smokers.	NR	NR
Akram et al. (2020)	*Lactobacillus rhamnosus PB01*, *Lactobacillus curvatus*, *Lactobacillus planatarum*, *Lactobacillus brevis*, *Pediococcus acidilactici*, *Bacillus subtilis*, *Bacillus megaterium*, *Bacillus pumulus spores*, *Lactobacillus reuteri*, and *Bifidobacterium lactis*	Tablet, toothpaste, lozenges, and chewing gum	2 wk to 9 mo	2 to 8 wk	Tooth	Not considered as a criterion	Cochrane Collaboration’s risk of bias tool CONSORT statement	*I*^2^ statistics and χ^2^ statistics Significant heterogeneity and limited available data may reduce the impact of these conclusions. Meta-analysis could not be performed for BOP due to significant heterogeneity (and use of different probiotics).	A total of 10 double-blind placebo-parallel RCTs were included. All studies showed that probiotic administration was effective in the treatment of gingivitis at follow-up. The mean percentage of BOP ranged from 11.87% to 21.7% in the probiotics group and from 15% to 33% in the placebo groups at follow-up, respectively. Considering the effects of *Lactobacillus reuteri*, the overall mean difference for GI (WMD = 0.48; 95% CI, 1.69–0.72; *P* = 0.42) and PI (WMD = 0.18; 95% CI, 0.23–0.61; *P* = 0.37) did not show any statistical significance between probiotic and placebo groups. Within the limitations of this study, the outcomes of this review show weak evidence to support the use of probiotics in mildly reducing inflammatory periodontal parameters in gingivitis.	NR	GRADE assessment
Gheisary et al. (2022)	*B. megaterium*, *B. pumulus*, *B. subtilis*, *B. bifidum*, *L. acidophilus*, *L. casei*, *L. rhamnosus*, *L. salivarius*, *Bifidobacterium*, *B. longum*, *L. bulgaricus*, *S. thermophilus*, *B. animalis*, *B. bifidum*, *L. delbrueckii*, *L. plantarum*, *S. boulardii*, *B. mesentericus*, *C. butyricum*, *L. sporogenes*, *S. faecalis*, *S. oralis*, *S. rattus*, *S. uberis*, *E. faecium*, *B. coagulans*, *L. curvatus*, *P. acidilactici*	Lozenge, capsule, tablet, powder, probiotic drink, probiotic-fortified food, toothpaste, mouthwash, spray, or subgingival delivery	Once to 3 mo	1 wk to 9 mo	Tooth	Not considered as a criterion	Cochrane Collaboration’s risk of bias tool	*I*^2^, which can be interpreted as low (25%), moderate (50%), or high (75%)	Of the 1,883 articles initially identified, 64 randomized clinical trials were included in this study. The results of this meta-analysis indicated statistically significant improvements after probiotic supplementation in the majority of the clinical outcomes in periodontal disease patients, including the plaque index (SMD = 0.557; 95% CI, 0.228–0.885), gingival index (SMD = 0.920; 95% CI, 0.426–1.414), probing pocket depth (SMD = 0.578; 95% CI, 0.365–0.790), clinical attachment level (SMD = 0.413; 95% CI, 0.262–0.563), bleeding on probing (SMD = 0.841; 95% CI, 0.479–1.20), gingival crevicular fluid volume (SMD = 0.568; 95% CI, 0.235–0.902), reduction in the subgingival periodontopathogen count of Pg (SMD = 0.402; 95% CI, 0.120–0.685), Fn (SMD = 0.392; 95% CI, 0.127–0.658), and Tf (SMD = 0.341; 95% CI, 0.050–0.633), and immunological markers MMP-8 (SMD = 0.819; 95% CI, 0.417–1.221) and IL-6 (SMD = 0.361; 95% CI, 0.079–0.644).	PPD = 0.509 (0.311–0.706), *I*^2^ = ? CAL = 0.413 (0.262–0.563), *I*^2^ = ? BOP = 0.598 (0.289–0.906), *I*^2^ = ? Micro Pg = 0.402 (0.120–0.685), *I*^2^ = ? Tf = 0.341 (0.050–0.633), *I*^2^ = ? Fn = 0.392 (–0.127 to 0.658), *I*^2^ = ?	NR

Aa, *Aggregatibacter actinomycetemcomitans*; BI, bleeding index; BOP, bleeding on probing; CAL, clinical attachment level; CFU, colony-forming units; CI, confidence interval; Fn, *Fusobacterium nucleatum;* GCF, gingival crevicular fluid; GI, gingival index; IL, interleukin; LB, *Lactobacillus;* LR, *Lactobacillus reuteri*; MD, mean difference; MMP, metalloproteinase; NR, not reported; NSPT, nonsurgical periodontal treatment; OR, odds ratio; Pg, *Porphyromonas gingivalis;* Pi, *Prevotella intermedia*; PI, plaque index; PD, periodontal diseases; PPD, periodontal probing depth/probing pocket depth; RCT, randomized controlled trial; SMD, standardized mean difference; SRP, scaling and root planning; Tf, *Tannerella forsythia;* WMD, weighted mean difference; d, day; mo, month; wk, week; y, year.

Fourteen SRs detected no clinically relevant effects of probiotic therapy as an adjuvant to SRP (Teughels et al. 2011; Yanine et al. 2013; Gruner et al. 2016; Jayaram et al. 2016; Akram et al. 2020; Barootchi et al. 2020; Donos et al. 2020; Gao et al. 2020; Ho et al. 2020; Silva et al. 2020; Abdulkareem et al. 2021; Corbella et al. 2021; Liu et al. 2022; Sayardoust et al. 2022). With regards to the quality assessment, 10 SRs had a low risk of bias (Yanine et al. 2013; Gruner et al. 2016; Akram et al. 2020; Barootchi et al. 2020; Donos et al. 2020; Gao et al. 2020; Ho et al. 2020; Silva et al. 2020; Corbella et al. 2021; Liu et al. 2022). Three had a moderate risk of bias (Teughels et al. 2011; Jayaram et al. 2016; Sayardoust et al. 2022) and 1 SR had high risk of bias (Abdulkareem et al. 2021).

#### Primary outcomes

##### Studies showing a positive effect of probiotic therapy as an adjuvant on periodontal probing depth

Fourteen SRs published data on the short-term effects of the probiotics on periodontal probing depth (PPD) reduction in both tooth (Martin-Cabezas et al. 2016; Matsubara et al. 2016; Priyanka et al. 2016; Ikram et al. 2018; Song and Liu 2020; Vives-Soler and Chimenos-Kustner 2020; Canut-Delgado et al. 2021; Hu et al. 2021; Mishra et al. 2021; Gheisary et al. 2022; Hardan et al. 2022; Ng et al. 2022) and implant substrates (Vives-Soler and Chimenos-Kustner 2020; Arbildo-Vega et al. 2021). *Lactobacillus reuteri* was the subspecies that showed the best effects on this clinical parameter (Priyanka et al. 2016; Arbildo-Vega et al. 2021). One SR showed a reduction in PPD with *Lactobacillus* treatment and no differences between coadjuvant treatment with *Streptococcus* probiotics and conventional mechanical treatment alone (Matsubara et al. 2016).

The outcomes on tooth substrate, from a 1 month follow-up, did not detect any clinical benefits (Ikram et al. 2018). Nine studies revealed a significant favorable response to SRP + probiotics in PPD reduction at the 3 months follow-up (Martin-Cabezas et al. 2016; Matsubara et al. 2016; Priyanka et al. 2016; Song and Liu 2020; Hu et al. 2021; Mishra et al. 2021; Gheisary et al. 2022; Hardan et al. 2022; Ng et al. 2022); this was more significant when pockets were divided according to depth (moderate and severe) (Martin-Cabezas et al. 2016; Vives-Soler and Chimenos-Kustner 2020; Canut-Delgado et al. 2021; Hu et al. 2021). However, SRs also suggested that mid-term results were not as favorable, with a less significant PPD reduction in the probiotic groups than in the control/placebo groups at the 6 months follow-up (Song and Liu 2020; Hu et al. 2021; Ng et al. 2022).

The outcomes on implant substrates showed PPD improvement in the probiotic groups, although not always achieving statistical significance (Arbildo-Vega et al. 2021). Moreover, PPD showed significant statistical heterogeneity among primary studies in the 3- and 6 months analyses.

Heterogeneity (*I*^2^) ranged from 63% (Gheisary et al. 2022) to 99% (Song and Liu 2020; Mishra et al. 2021) in the different studies. The lowest percentage was associated with a shorter follow-up period.

##### Studies showing no effect of probiotic therapy as an adjuvant on PPD

Eleven SRs reported no statistically significant short-term effects of using probiotics as an adjuvant on PPD reduction in both tooth(Teughels et al. 2011; Gruner et al. 2016;Jayaram et al. 2016; Donos et al. 2020; Ho et al. 2020; Abdulkareem et al. 2021; Corbella et al. 2021) and implant (Barootchi et al. 2020; Gao et al. 2020; Silva et al. 2020; Sayardoust et al. 2022) substrates. The overall effect of the coadjuvant therapy did not show a clinically relevant improvement on tooth substrates’ clinical parameters (Donos et al. 2020; Ho et al. 2020; Corbella et al. 2021). One SR that included both smokers and animal studies found a significant PPD improvement in smokers and no significant benefit in animals due to the lack of oral hygiene habits, respectively (Teughels et al. 2011). In mucositis and peri-implantitis, 4 SRs showed a PPD reduction of approximately 0.4 mm after treatment completion but did not indicate significant clinical effects of probiotic therapy (Barootchi et al. 2020; Gao et al. 2020; Silva et al. 2020; Sayardoust et al. 2022).

##### Studies showing a positive effect of probiotic therapy as an adjuvant on clinical attachment level

Ten SRs published findings on the short-term effect of adjuvant probiotics on clinical attachment level (CAL) gain in tooth substrates (Martin-Cabezas et al. 2016; Matsubara et al. 2016; Ikram et al. 2018; Song and Liu 2020; Canut-Delgado et al. 2021; Hu et al. 2021; Mishra et al. 2021; Gheisary et al. 2022; Hardan et al. 2022). Patients with chronic periodontitis under probiotic therapy adjuvant to SRP attained CAL gain in the 1- and 3 months follow-up (Ikram et al. 2018; Song and Liu 2020; Hu et al. 2021; Mishra et al. 2021; Gheisary et al. 2022; Hardan et al. 2022). This was more significant when pockets were divided according to depth (moderate [Martin-Cabezas et al. 2016; Matsubara et al. 2016] and severe [Martin-Cabezas et al. 2016; Matsubara et al. 2016]). There was no evidence of significant CAL gain after 6 months (Hu et al. 2021).

Heterogeneity ranged from 62% to 97% in the included studies at the 3- and 6-mo follow-ups, respectively.

##### Studies showing no effect of probiotic therapy as an adjuvant on CAL

Nine SRs reported found no effect on using probiotics as an adjuvant on the short-term effect on CAL gain in tooth substrates (Teughels et al. 2011; Yanine et al. 2013; Gruner et al. 2016; Jayaram et al. 2016; Priyanka et al. 2016; Donos et al. 2020; Ho et al. 2020; Abdulkareem et al. 2021; Corbella et al. 2021). Seven SRs found no significant differences in CAL in the groups with probiotic adjuvant therapy (Teughels et al. 2011; Yanine et al. 2013; Gruner et al. 2016; Priyanka et al. 2016; Donos et al. 2020; Ho et al. 2020; Corbella et al. 2021). One SR reported no significant differences in CAL gain due to including smokers and animals in the studies and not adjusting for smoking habits and oral hygiene (Teughels et al. 2011).

The results for CAL outcomes were coherent with PPD data, with a significant benefit observed at 3 months but not at 6 months (Ho et al. 2020; Corbella et al. 2021). SRs found a positive effect of probiotics at 12 months (Ho et al. 2020; Corbella et al. 2021). However, the heterogeneity for this outcome, although smaller than for the PPD outcome, was still considerable, ranging from 0% (Gruner et al. 2016) to 96% (Ho et al. 2020). Like PPD outcome, the subspecies *Lactobacillus reuteri* was the most effective in improving this clinical parameter than other probiotics (Corbella et al. 2021).

##### Studies showing a positive effect of probiotic therapy as an adjuvant on bleeding on probing

Twelve SRs reported on the short-term effects of probiotics as an adjuvant on bleeding on probing (BOP) reduction in both tooth (Gruner et al. 2016; Martin-Cabezas et al. 2016; Matsubara et al. 2016; Priyanka et al. 2016; Vives-Soler and Chimenos-Kustner 2020; Canut-Delgado et al. 2021; Hu et al. 2021; Gheisary et al. 2022; Hardan et al. 2022) and implant (Silva et al. 2020; Vives-Soler and Chimenos-Kustner 2020; Arbildo-Vega et al. 2021) substrates.

For the tooth substrate, BOP reduction was higher in groups under NSPT + probiotic therapy (Gruner et al. 2016; Martin-Cabezas et al. 2016; Matsubara et al. 2016; Priyanka et al. 2016; Vives-Soler and Chimenos-Kustner 2020; Canut-Delgado et al. 2021; Hu et al. 2021; Gheisary et al. 2022; Hardan et al. 2022), remaining stable in the first year of follow-up (Matsubara et al. 2016). Similar to PPD and CAL outcomes, significant BOP reduction was also demonstrated at 3 months, but the studies reporting outcomes at 6 months were conflicting (Hu et al. 2021). The BOP outcome for *Lactobacillus* was positive but not for *Streptococcus* (Matsubara et al. 2016). Moreover, BOP outcomes were better with the bleeding index of Saxton (Vives-Soler and Chimenos-Kustner 2020).

For the implant substrate, the differences were not significant between groups for all studies (Silva et al. 2020; Vives-Soler and Chimenos-Kustner 2020; Arbildo-Vega et al. 2021).

##### Studies showing no effect of probiotic therapy as an adjuvant on BOP

Ten SRs published outcomes showing no positive effect on the short-term effect on BOP reduction in both tooth (Teughels et al. 2011; Yanine et al. 2013; Jayaram et al. 2016; Akram et al. 2020; Barboza et al. 2020; Abdulkareem et al. 2021; Liu et al. 2022) and implant substrates (Barootchi et al. 2020; Gao et al. 2020; Sayardoust et al. 2022).

For the tooth substrate, the overall effect of the coadjuvant therapy did not show clinically significant differences in BOP (Teughels et al. 2011; Jayaram et al. 2016; Barboza et al. 2020; Liu et al. 2022). Yanine et al. (2013) found that this was related to different BOP measurements. The included SRs lacked clinical data on this outcome (Akram et al. 2020). There was also considerable heterogeneity, with 1 study reporting significant heterogeneity that compromised the performance of a meta-analysis (Akram et al. 2020) and another not reporting the existence of heterogeneity (Liu et al. 2022).

In the implant substrate, adjuvant probiotics did not significantly reduce BOP around the implant (Barootchi et al. 2020; Gao et al. 2020), even with the *Lactobacillus* probiotic species (Gao et al. 2020). One SR found a significant beneficial effect of probiotic therapy on BOP reduction (Sayardoust et al. 2022).

#### Secondary outcomes

##### Studies showing a positive effect of probiotic therapy as an adjuvant on microbiological analysis

Six SRs reported studies showing the positive effect of using probiotics as an adjuvant on reducing periodontal pathogen on at tooth substrates (Song and Liu 2020; Vives-Soler and Chimenos-Kustner 2020; Canut-Delgado et al. 2021; Mishra et al. 2021; Saïz et al. 2021; Gheisary et al. 2022) and implant (Saïz et al. 2021). The periodontal pathogen reduction was significantly higher in the groups under SRP + probiotic therapy (Song and Liu 2020; Vives-Soler and Chimenos-Kustner 2020; Canut-Delgado et al. 2021; Mishra et al. 2021; Saïz et al. 2021). The pathogens evaluated were *Aggregatibacter actinomycetemcomitans* in 4 SRs (Song and Liu 2020; Vives-Soler and Chimenos-Kustner 2020; Canut-Delgado et al. 2021; Mishra et al. 2021), *Porphyromonas gingivalis* in 5 SRs (Song and Liu 2020; Vives-Soler and Chimenos-Kustner 2020; Canut-Delgado et al. 2021; Mishra et al. 2021; Gheisary et al. 2022), *Prevotella intermedia* in 3 SRs (Song and Liu 2020; Vives-Soler and Chimenos-Kustner 2020; Canut-Delgado et al. 2021), *Treponema denticola* in 1 SR (Canut-Delgado et al. 2021), *Fusobacterium nucleatum* in 2 SRs (Canut-Delgado et al. 2021; Gheisary et al. 2022), and *Tannerella forsythia* in 1 SR (Gheisary et al. 2022). *P. gingivalis* reduction was observed at 12 weeks, while *A. actinomycetemcomitans* reduction occurred at 3 weeks of follow-up (Mishra et al. 2021). The heterogeneity for *A. actinomycetemcomitans* was moderate (0%–74%) and for *P. gingivalis* was substantial (96%–97%) (Mishra et al. 2021).

##### Studies showing no effect of probiotic therapy as an adjuvant on microbiological analysis

Eight SRs reported no short-term effects of adjuvant probiotics on periodontal pathogen reduction in both tooth substrates (Teughels et al. 2011; Gruner et al. 2016; Seminario-Amez et al. 2017; Ho et al. 2020; Liu et al. 2022; Ng et al. 2022) and implant substrates (Silva et al. 2020; Sayardoust et al. 2022). Most SRs did not identify significant differences when NSPT was combined with probiotics (Teughels et al. 2011; Gruner et al. 2016; Seminario-Amez et al. 2017; Ho et al. 2020; Liu et al. 2022; Ng et al. 2022). The pathogens evaluated were *A. actinomycetemcomitans* in 2 SRs (Gruner et al. 2016; Liu et al. 2022), *P. gingivalis* in 2 SRs (Gruner et al. 2016; Liu et al. 2022), *P. intermedia* in 2 SRs (Gruner et al. 2016; Liu et al. 2022), and *F. nucleatum* in 1 SR (Liu et al. 2022). No significant heterogeneity was found (Liu et al. 2022). The findings in mucositis and peri-implantitis showed a very limited effect of probiotics on the peri-implant microbiota (Silva et al. 2020; Sayardoust et al. 2022).

##### Influence of systemic diseases

Twenty-three SRs (Teughels et al. 2011; Gruner et al. 2016; Jayaram et al. 2016; Matsubara et al. 2016; Priyanka et al. 2016; Seminario-Amez et al. 2017; Ikram et al. 2018; Akram et al. 2020; Barboza et al. 2020; Barootchi et al. 2020; Donos et al. 2020; Gao et al. 2020; Ho et al. 2020; Vives-Soler and Chimenos-Kustner 2020; Abdulkareem et al. 2021; Arbildo-Vega et al. 2021; Corbella et al. 2021; Hu et al. 2021; Mishra et al. 2021; Saïz et al. 2021; Gheisary et al. 2022; Hardan et al. 2022; Sayardoust et al. 2022) did not consider the influence of systemic disease as an eligibility criterion while 7 SRs (Yanine et al. 2013; Martin-Cabezas et al. 2016; Silva et al. 2020; Song and Liu 2020; Canut-Delgado et al. 2021; Liu et al. 2022; Ng et al. 2022) considered it as an exclusion criterion on the recruitment phase. The only SR (Corbella et al. 2021) that divided the results by healthy and systemically compromised patients reported no relevant randomized controlled trial where NSPT was combined with adjuvant probiotic therapy in systemically compromised subjects.

Table 4 summarizes the findings of the included SRs.

**Table 4.** Summary of Findings.

Effects of probiotic therapy on periodontal and peri-implant treatments: An umbrella review						
Patients or population: Adult patients (≥18 y) diagnosed with periodontal disease and/or peri-implant disease Settings: Periodontology Intervention: Probiotic therapy^a^ Comparison: Conventional therapy^b^						
Outcomes	Total Number of Studies	Studies Advocating Probiotic Therapy’s Clinical Relevance		Studies Not Advocating Probiotic Therapy’s Clinical Relevance		Comments
		Tooth	Implant	Tooth	Implant	
PPD	26	13	2	7	4	Coherent evidence of favorability within the initial 3 mo, with the beneficial effect of probiotics diminishing in medium- to long-term follow-up.
CAL	19	10	0	9	0	Coherent evidence of favorability in the first and third months. Benefit at 6 mo still to be demonstrated.
BOP	23	10	3	7	3	Benefit at 6 mo still to be demonstrated.
Bone loss around teeth or implants	No clinical data					
Survival rate of implants	No clinical data					
Tooth loss	No clinical data					
Systemic diseases	1	—		1		8 studies consider this outcome as an exclusion criterion
Microbiological analysis	14	6		8		—

BOP, bleeding on probing; CAL, clinical attachment level; PPD, periodontal probing depth/probing pocket depth.

a Includes probiotic therapy alone or a combination of species (*Lactobacillus*, *Bifidobacterium*, *Streptococcus*, *Bacillus*, *Clostridium*, *Saccharomyces*, *Pediococcus*, and subspecies of each).

b Includes nonsurgical treatment, subgingival debridement, manual mechanical therapy, scaling, and root planning alone or with placebo.

## Discussion

### Summary of the Main Findings

This umbrella review included 30 SRs (Teughels et al. 2011; Yanine et al. 2013; Gruner et al. 2016; Jayaram et al. 2016; Martin-Cabezas et al. 2016; Matsubara et al. 2016; Priyanka et al. 2016; Seminario-Amez et al. 2017; Ikram et al. 2018; Akram et al. 2020; Barboza et al. 2020; Barootchi et al. 2020; Donos et al. 2020; Gao et al. 2020; Ho et al. 2020; Silva et al. 2020; Song and Liu 2020; Vives-Soler and Chimenos-Kustner 2020; Abdulkareem et al. 2021; Arbildo-Vega et al. 2021; Canut-Delgado et al. 2021; Corbella et al. 2021; Hu et al. 2021; Mishra et al. 2021; Saïz et al. 2021; Gheisary et al. 2022; Hardan et al. 2022; Liu et al. 2022; Ng et al. 2022; Sayardoust et al. 2022). A quantitative analysis of the results was not possible due to the high heterogeneity of clinical data.

Despite there being 30 previously published SRs, the evidence on the effectiveness of using probiotics as an adjuvant to conventional NSPT is still uncertain. In the included SRs, the short- and mid-term success of probiotics was determined, but their effect in the long term is still unclear. The SRs showed that long-term benefits were not evident at the 6 months evaluation (Song and Liu 2020; Hu et al. 2021; Ng et al. 2022). There is considerable heterogeneity in the variety of probiotic subspecies, probiotic regimens, and treatment protocols. However, a longer probiotic administration period seemed to be associated with prolonged maintenance of the probiotics’ effects on clinical parameters. The *Lactobacillus* species with *reuteri* subspecies produced the best improvement in the clinical parameters (Priyanka et al. 2016; Arbildo-Vega et al. 2021; Corbella et al. 2021).

### Limitations of the Review and Importance on Clinical Practice and Research

Since this umbrella review included SRs, its limitations are directly related to those of the included SRs and, subsequently, the corresponding primary studies. Eighty-one percent of SRs had been reported as per the recommended PRISMA guidelines, and 29% had a risk of bias between moderate and high. This can be attributed to certain common methodological limitations such as the lack of a definition of a standardized treatment protocol, the lack of knowledge of the most effective probiotic combinations, the most appropriate probiotic vehicle, and the frequency of administration, and thus the probiotics’ clinical benefits on clinical parameters must be interpreted with caution. The main reason for conflicting evidence among the included studies was the variety in probiotic subspecies, probiotic regimens, chronic periodontitis definitions in primary studies, treatment protocols, follow-up periods, inclusion criteria (like smoking habits), and sample sizes. These aspects compromise the heterogeneity among the included primary studies of the SRs, often preventing a meta-analysis of the results.

These limitations hinder probiotic use in clinical practice, despite potential benefits on periodontal health.

### Future Directions and/or Recommendations

The findings of this umbrella review highlight the need for future research in this field, focusing on RCTs with extended follow-up periods, 1 year at minimum, to better understand the sustained effects of probiotics. RCTs should strive for uniformity in the route of probiotic administration to ensure a consistent and commensurate basis for assessment; should engage in a comparative analysis to discern the advantages between protocols employing single probiotic species and those using a combination of probiotics, thereby ascertaining the most efficacious approach; and ought to standardize the methodologies for collecting clinical data related to periodontal outcomes, thereby enhancing the comparability and reliability of the obtained results.

## Conclusion

Based on the available evidence, the results are conflicting, and there can be no definitive conclusion for or against probiotics. Future studies controlling for all the confounding variables mentioned are needed.

## Author Contributions

C. Mendonça, contributed to conception, design, data acquisition, analysis, and interpretation, drafted the manuscript; D. Marques, contributed to conception, design, data interpretation, critically revised the manuscript; J. Silveira, J. Marques, contributed to conception, design, critically revised the manuscript; R.F. de Souza, contributed to data analysis, and interpretation, critically revised the manuscript; A. Mata, contributed to conception, design, data analysis, critically revised the manuscript. All authors have their final approval and agree to be accountable for all aspects of work.

## Supplemental Material

sj-pdf-1-jct-10.1177_23800844241240474 – Supplemental material for Effects of Probiotic Therapy on Periodontal and Peri-implant Treatments: An Umbrella Review Supplemental material, sj-pdf-1-jct-10.1177_23800844241240474 for Effects of Probiotic Therapy on Periodontal and Peri-implant Treatments: An Umbrella Review by C. Mendonça, D. Marques, J. Silveira, J. Marques, R.F. de Souza and A. Mata in JDR Clinical & Translational Research
