# Supplementary material for: Effects of Probiotic Therapy on Periodontal and Peri-implant Treatments: An Umbrella Review
Source: JDR Clin Trans Res. 2024 Nov 7;10(3):246–68. doi: 10.1177/23800844241240474 (PMC12166148; doi:10.1177/23800844241240474)
Supplement: sj-pdf-1-jct-10.1177_23800844241240474 – Supplemental material for Effects of Probiotic Therapy on Periodontal and Peri-implant Treatments: An Umbrella Review [file sj-pdf-1-jct-10.1177_23800844241240474.pdf]

Effects of probiotic therapy on periodontal treatments: an umbrella review

Carlota Duarte de Mendonça[1,2,3,4], Duarte Marques[1,2,3,5], João Silveira[1,2,3], Joana Faria Marques[1,2,3], Raphael de Souza[6], António Mata[1,2,3,4]

1. Biology and Oral Biochemistry Group, LIBPhys-FCT UID/FIS/04559/2013, Faculty of Dental Medicine, University of Lisbon, 1649-003, Lisbon, Portugal.
2. Center for Evidence-Based Dental Medicine, Faculty of Dental Medicine, University of Lisbon, 1649-003, Lisbon, Portugal.
3. Faculty of Dental Medicine, University of Lisbon, 1649-003, Lisbon, Portugal.
4. Hugo Madeira Clinic - Advanced Aesthetics and Implantology, Av. Casal Ribeiro, No50, 1000-093, Lisbon, Portugal.
5. Institute of Implantology, Av. Columbano Bordalo Pinheiro 50, 1070-064 Lisbon, Portugal.
6. Faculty of Dental Medicine and Oral Health Sciences, 3640, University St., # M/65A, Montreal (Quebec) Canada H3A 2B2.

Supplementary Material

Appendix 1: PRISMA checklist.

Appendix 2: Information Sources and Search for PICO.

Appendix 3: Flow Diagram for PICO.

Appendix 4: Excluded studies after full-text analysis and main reason for exclusion for PICO.

Appendix 5: Risk of bias in studies

Appendix 1: PRISMA checklist

Table 5 - PRISMA checklist

Section and Topic	Item #	Checklist item	Location where item is reported
**TITLE**			
Title	1	Identify the report as a systematic review.	1
**ABSTRACT**			
Abstract	2	See the PRISMA 2020 for Abstracts checklist.	1
**INTRODUCTION**			
Rationale	3	Describe the rationale for the review in the context of existing knowledge.	2
Objectives	4	Provide an explicit statement of the objective(s) or question(s) the review addresses.	2
**METHODS**			
Eligibility criteria	5	Specify the inclusion and exclusion criteria for the review and how studies were grouped for the syntheses.	2
Information sources	6	Specify all databases, registers, websites, organisations, reference lists and other sources searched or consulted to identify studies. Specify the date when each source was last searched or consulted.	2
Search strategy	7	Present the full search strategies for all databases, registers and websites, including any filters and limits used.	2
Selection process	8	Specify the methods used to decide whether a study met the inclusion criteria of the review, including how many reviewers screened each record and each report retrieved, whether they worked independently, and if applicable, details of automation tools used in the process.	3
Data collection process	9	Specify the methods used to collect data from reports, including how many reviewers collected data from each report, whether they worked independently, any processes for obtaining or confirming data from study investigators, and if applicable, details of automation tools used in the process.	3
Data items	10a	List and define all outcomes for which data were sought. Specify whether all results that were compatible with each outcome domain in each study were sought (e.g. for all measures, time points, analyses), and if not, the methods used to decide which results to collect.	-
	10b	List and define all other variables for which data were sought (e.g. participant and intervention characteristics, funding sources). Describe any assumptions made about any missing or unclear information.	-
Study risk of bias assessment	11	Specify the methods used to assess risk of bias in the included studies, including details of the tool(s) used, how many reviewers assessed each study and whether they worked independently, and if applicable, details of automation tools used in the process.	3
Effect measures	12	Specify for each outcome the effect measure(s) (e.g. risk ratio, mean difference) used in the synthesis or presentation of results.	3
Synthesis methods	13a	Describe the processes used to decide which studies were eligible for each synthesis (e.g. tabulating the study intervention characteristics and comparing against the planned groups for each synthesis (item #5)).	3
	13b	Describe any methods required to prepare the data for presentation or synthesis, such as handling of missing summary statistics, or data conversions.	-
	13c	Describe any methods used to tabulate or visually display results of individual studies and syntheses.	-
	13d	Describe any methods used to synthesize results and provide a rationale for the choice(s). If meta-analysis was performed, describe the model(s), method(s) to identify the presence and extent of statistical heterogeneity, and software package(s) used.	-
	13e	Describe any methods used to explore possible causes of heterogeneity among study results (e.g. subgroup analysis, meta-regression).	-

Section and Topic	Item #	Checklist item	Location where item is reported
	13f	Describe any sensitivity analyses conducted to assess robustness of the synthesized results.	-
Reporting bias assessment	14	Describe any methods used to assess risk of bias due to missing results in a synthesis (arising from reporting biases).	-
Certainty assessment	15	Describe any methods used to assess certainty (or confidence) in the body of evidence for an outcome.	-
**RESULTS**			
Study selection	16a	Describe the results of the search and selection process, from the number of records identified in the search to the number of studies included in the review, ideally using a flow diagram.	3
	16b	Cite studies that might appear to meet the inclusion criteria, but which were excluded, and explain why they were excluded.	3
Study characteristics	17	Cite each included study and present its characteristics.	3
Risk of bias in studies	18	Present assessments of risk of bias for each included study.	9
Results of individual studies	19	For all outcomes, present, for each study: (a) summary statistics for each group (where appropriate) and (b) an effect estimate and its precision (e.g. confidence/credible interval), ideally using structured tables or plots.	9
Results of syntheses	20a	For each synthesis, briefly summarise the characteristics and risk of bias among contributing studies.	9
	20b	Present results of all statistical syntheses conducted. If meta-analysis was done, present for each the summary estimate and its precision (e.g. confidence/credible interval) and measures of statistical heterogeneity. If comparing groups, describe the direction of the effect.	-
	20c	Present results of all investigations of possible causes of heterogeneity among study results.	-
	20d	Present results of all sensitivity analyses conducted to assess the robustness of the synthesized results.	-
Reporting biases	21	Present assessments of risk of bias due to missing results (arising from reporting biases) for each synthesis assessed.	-
Certainty of evidence	22	Present assessments of certainty (or confidence) in the body of evidence for each outcome assessed.	-
**DISCUSSION**			
Discussion	23a	Provide a general interpretation of the results in the context of other evidence.	20
	23b	Discuss any limitations of the evidence included in the review.	21
	23c	Discuss any limitations of the review processes used.	21
	23d	Discuss implications of the results for practice, policy, and future research.	21
**OTHER INFORMATION**			
Registration and protocol	24a	Provide registration information for the review, including register name and registration number, or state that the review was not registered.	2
	24b	Indicate where the review protocol can be accessed, or state that a protocol was not prepared.	-
	24c	Describe and explain any amendments to information provided at registration or in the protocol.	-
Support	25	Describe sources of financial or non-financial support for the review, and the role of the funders or sponsors in the review.	21
Competing interests	26	Declare any competing interests of review authors.	21

Section and Topic	Item #	Checklist item	Location where item is reported
Availability of data, code and other materials	27	Report which of the following are publicly available and where they can be found: template data collection forms; data extracted from included studies; data used for all analyses; analytic code; any other materials used in the review.	-

From: Page MJ, McKenzie JE, Bossuyt PM, Boutron I, Hoffmann TC, Mulrow CD, et al. The PRISMA 2020 statement: an updated guideline for reporting systematic reviews. BMJ 2021;372:n71. doi: 10.1136/bmj.n71

For more information, visit: http://www.prisma-statement.org/

Appendix 2: Information Sources and Search for PICO

Algorithm MEDLINE (via PubMed)

1(“gingivitis” [MeSH Terms] OR “periodontal disease” [MeSH Terms] OR “periodontitis” [MeSH Terms] OR “alveolar bone loss” [MeSH Terms] OR “Periodontic*” [MeSH Terms] OR “Peri-Implantitis" [MeSH Terms]

2 (“oral conditions” [Text Word] OR “dental plaque” [Text Word] OR “oral hygiene” [Text Word] OR “tooth diseases” [All fields])

3 (#1) OR (#2)

4 ("probiotic*” [MeSH Terms] OR “probiotic*” [All Fields])) OR “prebiotic*” [All Fields] OR “symbiotic*” [All Fields]]

5 “Bifidobacterium"[All Fields] OR "Lactobacillus"[All Fields] OR “Streptococcus”[All Fields] OR “Bacillus” [All Fields]

6 (#4) OR (#5)

7 (#3) AND (#6)

Appendix 3: Flow Diagram for PICO

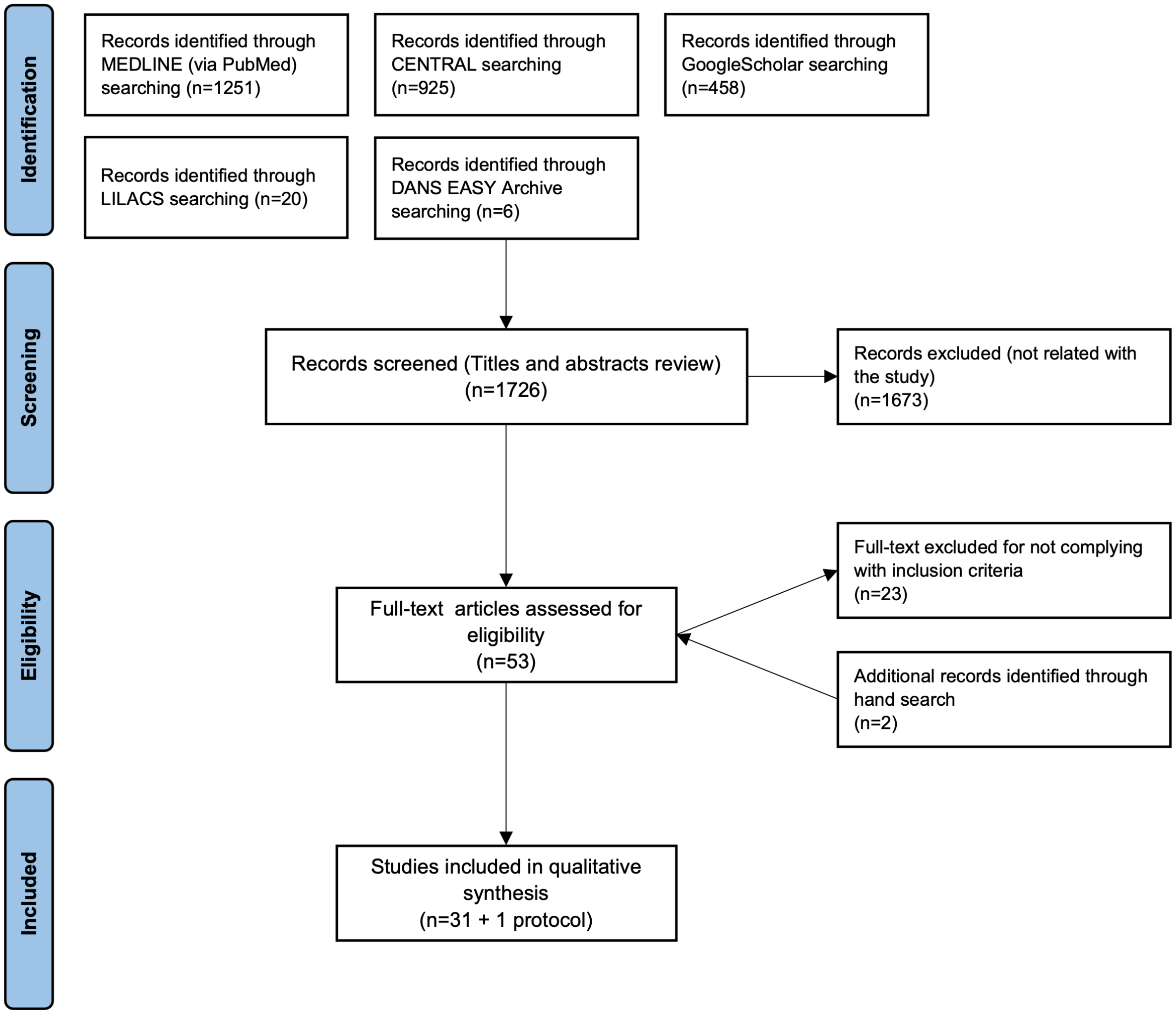

Figure 1 - Flow chart summarizing the search strategy and results.

Appendix 4: Excluded studies after full-text analysis and main reason for exclusion (n=23) for PICO

Table 6 - Table of excluded studies(1) methodology not compatible with the systematic review according to the Cochrane Handbook for Systematic Reviews of Interventions definition (Higgins and Green 2011), (2) evaluating illnesses other than periodontal diseases, (3) including healthy periodontal patients, and (4) evaluating therapies different than probiotics.

Author (year)	Reason for exclusion
**Laleman et al. (2015)**	1
**Teughels et al. (2008)**	1
**Allaker et al. (2017)**	1
**Rondanelli et al. (2017)**	1
**Agarwal et al. (2015)**	1
**Shi et al. (2016)**	1
**Didari et al. (2014)**	2
**Zupancic et al. (2017)**	2
**Bustamante et al. (2020)**	1
**Emamie et al. (2020)**	2
**Kumar et al. (2017)**	1
**Nadelman et al. (2018)**	3
**Saraf et al. (2010)**	1
**Lawande et al. (2012)**	1
**Zhang et al. (2021)**	2
**Guerra et al. (2018)**	2
**Jørgensen et al. (2016)**	1
**Lang et al. (2019)**	2
**Morales et al. (2017)**	1
**Tamayo et al. (2008)**	2
**Dhingra et al. (2012)**	1/2
**Cionca et al. (2009)**	4
**Devine et al. (2009)**	1/2

Appendix 5: Risk of bias in studies

Table 7 - Risk of bias in studies according to JBI Critical Appraisal tools for use in the JBI Systematic Reviews - Checklist for Systematic Reviews.

Critical Appraisal tools for use in JBI Systematic Reviews												
**Author year**	1. Is the review question clearly and explicitly stated?	2.Were the inclusion criteria appropriate for the review question?	3. Was the search strategy appropriate?	4. Were the sources and resources used to search for studies adequate?	5. Were the criteria for appraising studies appropriate?	6. Was critical appraisal conducted by two or more reviewers independently?	7. Were there methods to minimize errors in data extraction?	8.Were the methods used to combine studies appropriate?	9. Was the likelihood of publication bias assessed?	10. Were recommendations for policy and/or practice supported by the reported data?	11. Were the specific directives for new research appropriate?	Comments (Including reason for exclusion)
**Alexandre Pires Silva (2020)(Silva et al. 2020)**	yes	yes	unclear	yes	yes	yes	unclear	yes	not applicable	yes	yes	3. very broad keywords<br>7. Not mentioned, but they mention three reviewers for study selection and two for quality assessment
**Ali Abdulkareem (2022)(Abdulkareem et al. 2021)**	yes	unclear	unclear	unclear	no	no	unclear	yes	not applicable	yes	yes	
**Anna Vives-Soler (2020)(Vives-Soler and Chimenos-Kustner 2020)**	yes	unclear	no	no	yes	unclear	yes	yes	not applicable	yes	yes	3. Only included RCTs in English.<br>3. No access to the algorithm, very broad keywords<br>4. Only used Medline as database.<br>6. Not reported, but articles was independently screened by two reviewers
**Arbildo-Veja (2021)(Arbildo-Vega et al. 2021)**	yes	yes	unclear	yes	yes	yes	yes	yes	yes	yes	yes	3. No access to the algorithm
**D Song (2020)(Song and Liu 2020)**	yes	yes	unclear	yes	yes	yes	yes	no	no	yes	yes	3. Algorithm doesn't include MeshTerms<br>8. They use a fixed model for MA when I~2>70%<br>9. Doesn't mention this aspect on the review!<br>On search strategy they don't include time limit, but in the inclusion criteria only considered RCT published in the last 10 years.
**Daoyong Hu (2021)(Hu et al. 2021)**	yes	yes	yes	yes	yes	yes	yes	yes	yes	yes	yes	
**Deborah Gruner (2016)(Gruner et al. 2016)**	yes	yes	unclear	yes	yes	unclear	yes	yes	yes	yes	yes	3. Algorithm doesn't include MeshTerms<br>6. Not reported, but in the other sections data was collected independently by two reviewers
**Eliane Barboza (2020)(Barboza et al. 2020)**	yes	yes	yes	yes	yes	yes	yes	yes	not applicable	yes	yes	
**Ethan Ng (2021)(Ng et al. 2022)**	yes	yes	no	yes	yes	yes	yes	yes	no	yes	yes	2. Only included RCTs in English<br>9. Doesn't mention this aspect on the review!
**Huiqing Gou (2020)(Gou et al. 2020)**	yes	yes	yes	yes	yes	yes	yes	yes	yes	yes	yes	

**Jiaxuan Liu (2021)(Liu et al. 2022)**	yes	yes	yes	yes	yes	yes	yes	unclear	yes	yes	yes	8. Doesn't present the forest plots for the measure outcomes. But specify the model used in Methods.
**Jinxia Gao (2020)(Gao et al. 2020)**	yes	yes	yes	yes	yes	yes	yes	yes	yes	yes	yes	
**Kakarla Priyanka (2016)(Priyanka et al. 2016)**	yes	yes	yes	yes	yes	unclear	unclear	unclear	not applicable	yes	yes	6/7. doesn't specify<br>8. doesn't justify why they only did qualitative synthesis
**Louis Hardan (2022)(Hardan et al. 2022)**	yes	yes	yes	yes	yes	yes	unclear	yes	unclear	yes	yes	
**Maria Seminario-Amez (2019)(Seminario-Amez et al. 2017)**	unclear	yes	unclear	no	no	unclear	unclear	yes	not applicable	yes	yes	1. "The aim of this article is to review the published literature with the purpose of knowing the importance of using probiotics as a preventive and therapeutic method for oral infectious diseases management."<br>3. No access to the algorithm, very broad keywords<br>4. Only use Pubmed for the research<br>5. Only use critical appraisal tools for the evaluation of RCTs, and they also include a systematic review and 2 metanalysis<br>6. Not reported<br>7. Not reported
**N. Canut-Delgado, (2021)(Canut-Delgado et al. 2021),**	yes	yes	no	yes	yes	unclear	unclear	unclear	not applicable	yes	yes	3. very broad keywords; limit to 10 years<br>6/7. doesn't specify<br>8. doesn't justify why they only did qualitative synthesis
**Nicolás Yanine (2013)(Yanine et al. 2013)**	yes	yes	yes	yes	yes	unclear	yes	yes	not applicable	yes	yes	6. They wrote "we" but when exist two specific independent reviewers it is mentioned
**Nikolaos Donos (2020)(Donos et al. 2020)**	yes	yes	unclear	yes	yes	no	yes	yes	yes	yes	yes	3. No access to the algorithm, very broad keywords<br>6. "The quality of the included studies was assessed using the Cochrane Risk of Bias Tool for randomized clinical trials (RoB 2) (updated on 15 March 2019) by one calibrated examiner (NB) and 15% of the studies were also assessed by a second examiner (EC) to ensure agreement on the scoring system"
**Perrine Saïz (2021)(Saïz et al. 2021)**	yes	yes	yes	yes	no	no	unclear	yes	not applicable	yes	yes	
**Praveen Jayaram (2016)(Jayaram et al. 2016)**	yes	no	no	yes	yes	unclear	unclear	yes	not applicable	yes	yes	2. Only included RCTs in English<br>3. very broad keywords<br>6. not reported<br>7. not reported
**Rodrigo Martin-Cabezas (2016)(Martin-Cabezas et al. 2016)**	yes	yes	no	yes	yes	yes	yes	yes	yes	yes	yes	3. "Only articles published in English language have been considered."

**Sana Ikram (2018)(Ikram et al. 2018)**	yes	yes	no	yes	yes	unclear	yes	yes	yes	yes	yes	2. Only included RCTs in English<br>6. Not reported, but articles was independently screened by two reviewers and data extraction also
**Shariel Sayardoust (2022)(Sayardoust et al. 2022)**	yes	yes	yes	unclear	yes	yes	unclear	unclear	yes	yes	yes	
**Shayan Barootchi (2020)(Barootchi et al. 2020)**	yes	yes	yes	yes	yes	yes	yes	yes	yes	yes	yes	
**Shreetika Mishra (2021)(Mishra et al. 2021)**	yes	yes	yes	yes	yes	yes	yes	no	unclear	yes	no	8. Ignore heterogeneity (MA with fixed effect model, when I^2>70%).
**Stefano Corbella (2021)(Corbella et al. 2021)**	yes	yes	yes	yes	yes	yes	yes	yes	yes	yes	yes	
**Sze Nga Ho (2020)(Ho et al. 2020)**	yes	yes	no	yes	yes	yes	yes	yes	no	yes	yes	2. Only included RCTs in English<br>3. Tentar aceder ao apêndice com o algoritmo na FMDUL - via eduroam<br>9. "No publication bias was assessed given the limited number of studies."
**Victor Haruo Matsubara (2016) (Matsubara et al. 2016)**	yes	yes	unclear	yes	yes	unclear	yes	yes	not applicable	yes	yes	3. Algorithm doesn't include MeshTerms<br>6. Not reported, but articles was independently screened by two reviewers
**Wim Teughels (2011)(Teughels et al. 2011)**	yes	no	yes	yes	no	no	yes	yes	not applicable	yes	yes	2. PICO format for inclusion criteria are not well specified.
**Zohaib Akram (2020)(Akram et al. 2020)**	yes	yes	no	yes	yes	unclear	yes	yes	yes	yes	yes	2. Only included RCTs in English<br>3. Algorithm doesn't include MeshTerms<br>6. Not reported, but articles was independently screened by two reviewers
**Zohre Gheisary (2022)(Gheisary et al. 2022)**	yes	yes	yes	unclear	yes	yes	yes	yes	yes	yes	yes	4. Only 3 databases and do not count with grey literature

RoB: risk of bias; MA: meta-analysis; RCT: randomized controlled trials; RCTs: randomized controlled trials;

Table 8 - Final score of risk of bias.

Author_year	"Yes" answer (%)	Final classification
**Abdulkareem_2022**	36.36	High
**Akram_2020**	81.82	Low
**Arbildo-Vega_2021**	90.91	Low
**Barboza_2020**	90.91	Low
**Barootchi_2020**	100	Low
**Canut-Delgado_2021**	54.55	Moderate
**Corbella_2021**	100	Low
**Donos_2020**	81.82	Low
**Gao_2020**	100	Low
**Gheisary_2022**	90.91	Low
**Gou_2020**	100	Low
**Gruner_2016**	81.82	Low
**Hardan_2022**	81.82	Low
**Ho_2021**	81.82	Low
**Hu_2021**	100	Low
**Ikram_2018**	81.82	Low
**Jayaram_2016**	54.55	Moderate
**Liu_2021**	90.91	Low
**Martin-Cabezas_2016**	90.91	Low
**Matsubara_2016**	72.73	Low
**Mishra_2021**	72.73	Low
**Nga_2020**	81.82	Low
**Priyanka_2016**	63.64	Moderate
**Saïz_2021**	63.64	Moderate
**Sayrdoust_2022**	63.64	Moderate
**Seminario-Amez_2019**	36.36	High
**Silva_2020**	72.73	Low
**Song_2020**	72.73	Low
**Teughels_2011**	63.64	Moderate
**Vives-Soler_2020**	54.55	Moderate
**Yanine_2013**	81.82	Low

22 studies with a final classification of "Low"

2 study with a final classification of "High"

7 studies with a final classification of "Moderate"

Table 9 - Risk of bias in the randomized controlled trials included in the included systematic reviews

Author year	Appraisal instruments used	Appraisal rating
**Alexandre Pires Silva (2020)(Silva et al. 2020)**	CONSORT statement<br><br>Cochrane Collaboration group	**Risk of Bias**<br>There was complete agreement for the overall risk of bias for all studies evaluated. Quality assessment of the studies (Table 3) revealed that three studies showed a high risk of bias toward one or more domains, as they presented no adequate randomization (Tada et al., 2018; Peña et al., 2018); or showed no blinding of evaluators/assessors (Alqahtani et al., 2019). Two of the studies analyzed presented low risk of bias (Galofré et al., 2018; Hallström et al., 2015).
**Ali Abdulkareem (2022)(Abdulkareem et al. 2021)**	-	-
**Anna Vives-Soler (2020)(Vives-Soler and Chimenos-Kustner 2020)**	**Risk of Bias Assessment -** Cochrane Handbook for Systematic Reviews of Interventions	**Risk of Bias Assessment -** The estimated potential risk of bias was considered to be "low" in 6 studies (3,7,9-12), moderate in two (8,13) and high in Vivekananda's (14) study. Studies were defined as low risk of bias if all criteria were met. When missing one of these criteria, the study was classified as moderate, and it resulted in a high potential risk when two or more were missing.
**Arbildo-Veja (2021)(Arbildo-Vega et al. 2021)**	**Risk of bias assessment -** Cochrane tool for randomized studies	**Risk of bias -** Three studies showed high risk of bias and the other three studies showed low risk of bias (Fig. 2).
**D Song (2020)(Song and Liu 2020)**	**Risk of bias assessment -** Consolidated Standards of Reporting Trials statement (Cochrane Handbook for Systematic Reviews of Interventions)	**Risk ob bias -** the risk of bias was considered high in four RCT assessed. Five RCT estimated the sample size, reported masking of assessor(s), and methods of allocation concealment. All studies presented appropriate statistical analysis and description of withdrawals and dropouts.
**Daoyong Hu (2021)(Hu et al. 2021)**	**Cochrane Collaboration risk of bias tool for RCTs** (risk of bias)<br>**Egger's linear regression**	The risk of bias in individual studies was summarized in Appendix S2. 20 studies (10-12 , 17 , 20-31 , 33-36) were considered to have a low risk of bias in all the categories. Four studies (16, 18, 19, 23 , 32) failed to clarify the randomization procedure and blinding methods.
**Deborah Gruner (2016)(Gruner et al. 2016)**	**Trial sequential analysis** (TSA 0.9 (Copenhagen Trial Unit, Copenhagen, Denmark); **GRADE tool** (Grade Profiler 3.6) **Egger-test** and **Funnel plot**.<br>**Cochrane Collaboration risk of bias tool for RCTs (risk of bias)**	**Risk of Bias -** From all studies, 8 had low, 34 unclear and 8 high risk of bias (Tab. S4). Most often, allocation concealment (37), sequence generation (26) and blinding of personnel (13) were not sufficiently described or not satisfyingly performed. Cross-over studies used wash-out periods between 2 and 6 weeks, one study did not report such periods at all (31). The effects of risk of bias were considered serious.<br>**Evidence Grading -** Calculated diversity-adjusted required information sizes (Tab. 1) were reached for SM numbers, and in reach for PPD, BOP, and CAL. However, our findings for caries experience and periodontal disease (PPD, CAL) were inconsistent, as were findings for numbers of SM, LB, periodontal pathogens (AA, PG, PrI), and PI. This inconsistency was considered serious. Only for SM, BOP, GI and the numbers of new/non-arrested lesions, consistency was confirmed. For all outcomes, serious imprecision was found. Publication bias was suspected for several outcomes, too. Overall, the evidence supporting probiotic therapy was graded as very low for all outcomes; the confidence in any of the calculated effect estimates is thus very limited.
**Eliane Barboza (2020)(Barboza et al. 2020)**	**Risk of bias assessment -** Cochrane Collaboration's tool	**Risk of bias assessment -** The quality analysis of RCTs and CCT included in the study are shown in Table 3. Five studies showed a low risk of bias,9,20-23 two studies20,22 met all the criteria described in the Cochrane Collaboration's tool,14 and three studies scored negatively,9,21,23 one in each question. No study used the CONSORT statement guidelines.24
**Ethan Ng (2021)(Ng et al. 2022)**	**Risk of bias -** Cochrane Handbook	**Risk of bias -** Most studies had an overall low risk of bias, two had some concerns, and one was at high risk. A j coefficient of 1.00 (95% CI 1.00–1.00) for overall risk of bias was found, indicating perfect agreement in quality assessment. Seven studies received industry support, although it was reported that the company was not involved in data management.
**Huiqing Gou (2020)(Gou et al. 2020)**	**Risk of bias assessment -** Cochrane Reviewers' Handbook	**Risk of bias assessment -** All included studies in this systematic review were performed quality analysis according to the Cochrane Reviewers' Handbook (Higgins & Green 2011). All

		the included studies were doubled- blinded RCTs, and adopted computer-based randomization program, block randomization and randomization table. The allocation concealment, the shape of probiotics and placebo were identical envelopes, bottles, containers, and mouthwashes in all the included studies. All the outcome evaluators did not know the therapy protocol. Selective reporting and conflict of interests were not found in all studies [25-35]. Three researches [30, 33, 35] had lost interviews, one [30] was defined as unclear risk with the rate of follow-up below 10%, two were defined as high risk with the rate of follow-up more than 20% which could contribute to attribution bias and reporting bias [33, 35] (Figure 7). Overall, the literature included in this study was of good quality.
**Jiaxuan Liu (2021)(Liu et al. 2022)**	**Risk of bias assessment -** Cochrane Reviewers' Handbook.	**Risk of bias assessment -** Six articles did not clearly report their randomized generation method, two articles did not prove whether allocation concealment was implemented, and one was a single-blind trial. According to the Cochrane Risk Assessment tool, all these articles were assessed as high risk and the remaining were assessed as low risk. Selective reporting and other biases were rated as low-risk biases in all the studies, because sufficient details were provided to assess and pre-determine the study outcomes (Figures 2 and 3).
**Jinxia Gao (2020)(Gao et al. 2020)**	**Cochrane Collaboration's Risk of Bias tool** (methodological quality); **GRADE tool** (quality of evidence)	**Risk of Bias -** The assessment of risk of bias revealed that 5 studies had a low risk of bias(22,23,26,28). Two showed a high risk for incomplete outcome data(15,24). One reported a dropout of 3 patients in the probiotic group in the last week of the study period (24) while another study had a dropout of 1 patient in the control group in the last day (15). The results of risk of bias are illustrated in Figure 2 and Figure 3.<br>**GRADE assessment -** revealed that the accumulated quality of evidence was judged low for PPD of peri-implant mucositis more than 2 months after treatment termination and moderate for other parameters. The GRADE evidence levels are shown in Table 2.
**Kakarla Priyanka (2016)(Priyanka et al. 2016)**	**Risk of bias assessment -** Cochrane Collaboration tool	**Risk of bias assessment -** we classified 9 articles as having a low risk of bias and 6 articles as having a high risk of bias. Table 4 shows the domain in which the trails were judged to have the high risk of bias.
**Louis Hardan (2022)(Hardan et al. 2022)**	**Cochrane risk of bias tool for randomized clinical trials**	When analyzing the risk of bias, most studies were not at a high risk of bias except for the parameter related to reporting bias and other bias (protocol recorded at CONSORT or ClinicalTrials). The selection, performance, and detection of bias due to incomplete data were those which presented a low risk of bias.
**Maria Seminario-Amez (2019)(Seminario-Amez et al. 2017)**	**Quality of RCT's -** Jadad scale was used to assess the high quality of RCTs.	**Quality of RCT's -** Jadad qualificaiton is presented on Table 1 for included RCT's: 9 were rated "high quality" (two with rating 4 - *18, 22* - and seven with rating 5 - *27, 20, 28, 25, 23 ,24, 19*); 3 were rated "good quality" (all rated with 3 - *21, 26, 29*).
**N. Canut-Delgado, (2021)(Canut-Delgado et al. 2021),**	**Risk of bias assessment -** Cochrane Handbook for Systematic Reviews of Interventions	**Risk of bias assessment -** None of the studies reported included smokers, patients with systemic diseases, or pregnant women, or patients who had received antibiotics during the previous 6 months. However, Shimauchi et al. compared the non-smoking test and placebo groups with a separate group of smokers, divided also into placebo and test groups34. After analyzing the quality of the trials using the Cochrane Handbook for Systematic Reviews of Interventions (version 5.1.0), all trials analyzed were considered to be at low risk of bias, except for the Szkaradkiewicz et al. trial, which has a low/unclear risk for not reporting how the random sequence, allocation concealment and blinding of patients were performed 7 (table 2).
**Nicolás Yanine (2013)(Yanine et al. 2013)**	**Cochrane Collaboration's Risk of Bias tool** (risk of bias)	**Risk of Bias -** The four studies were subjected to critical analysis following the Cochrane Collaboration tool for evaluating the risk of bias, and we classified two articles as having a low risk of bias [10, 12] and two articles as having a high risk of bias [13, 14] (Table 4). The domain in which the trials were judged to have the lower risk of bias was "randomization". All the studies reported the randomization method properly. In contrast, the domain classified as having the higher risk of bias was "free of other sources of bias". The four studies were<br>funded by private laboratories. Other important sources of bias were the short follow-up period [13], experimentally

		induced gingivitis, and samples conformed by dental and medical students [14].
**Nikolaos Donos (2020)(Donos et al. 2020)**	**Assessment of Heterogeneity -** Cochran's test for heterogeneity<br><br>**Assessment of Reporting Bias -** Egger ́s bias<br><br>**Risk of Bias Assessment -** Cochrane Risk of Bias Tool for randomized clinical trials (RoB 2)	Assessment of Heterogeneity - low (25-50%), moderate (51-75%), or high (>75%) (Higgins et al., 2003).<br><br>Assessment of Reporting Bias - funnel plot asymmetry<br><br>**Risk of Bias Assessment -** Each study was judged as at low, moderate, high, or unclear risk-of-bias on the basis of five domains: (1) bias arising from the randomization process; (2) bias due to deviations from intended interventions; (3) bias due to missing outcome data; (4) bias in measurement of the outcome; (5) bias in selection of the reported result.
**Perrine Saïz (2021)(Saïz et al. 2021)**	**-**	-
**Praveen Jayaram (2016)(Jayaram et al. 2016)**	**Quality of repoorting -** CONSORT checklist	**CONSORT -** Of the 14 studies reviewed for the CONSORT guidelines, two of them fulfilled all the criteria. One of the parameters that were poorly addressed in the CONSORT checklist was registration of the trial in the trial registry. Only one of the studies were registered in the trial registry. Some of the trials did not mention the dates of recruitment of patients and follow-up, while others did not mention how randomization and blinding/concealment were done. The results of the application of CONSORT guidelines are given in Table 1. Many studies also failed to address how the randomization, allocation, concealment and blinding were performed during the course of the study. Not mentioning these items on the checklist may lead to an increased risk of selective reporting. Many of the newer studies addressed most of the items on the CONSORT checklist.
**Rodrigo Martin-Cabezas (2016)(Martin-Cabezas et al. 2016)**	**Cochrane Reviewers' Handbook (Higgins & Green 2011)** (risk of bias); **Review Manager** (Version 5.2. The Cochrane Collaboration, 2013, Oxford, UK)	**Risk of Bias -** table 2 and 3 (everything was ratted as low risk of bias).
**Sana Ikram (2018)(Ikram et al. 2018)**	**Risk of bias assessment -** Consolidated Standards of Reporting Trials statement (Cochrane Handbook for Systematic Reviews of Interventions)	**Risk of bias -** The risk of bias was considered high in four RCT assessed. Five RCT estimated the sample size, reported masking of assessor(s), and methods of allocation concealment. All studies presented appropriate statistical analysis and description of withdrawals and dropouts.
**Shariel Sayardoust (2022)(Sayardoust et al. 2022)**	**Cochrane Handbook for Systematic Reviews of Interventions to assess the risk of bias (Higgins and Altman, 2008) associated with concealment of allocation, randomization, blinding of outcome assessor, and blinding of patients in the studies.**	Four out of the 7 studies revealed low bias (Flichy-Fernández et al., 2015; Hallström et al., 2016; Galofré et al., 2018; Laleman et al., 2020). Selection bias was noted in the remaining 3 studies (Tada et al., 2018; Lauritano et al., 2019; Peña et al., 2019).
**Shayan Barootchi (2020)(Barootchi et al. 2020)**	**Risk of bias assessment.** Cochrane risk of bias tool for RCTs	**Risk of bias -** The results of the bias analysis for the RCTs are described in the Supplementary Table 2. Overall, eight articles were considered as having a high risk of bias14,16,29-34, three were categorised as show- ing a moderate risk of bias35-37, and three studies demonstrated a low risk of bias38-40.
**Shreetika Mishra (2021)(Mishra et al. 2021)**	**Risk of bias assessment -** Cochrane Handbook for systematic review	**Risk of bias -** Overall a low risk of bias was reported for the included studies for most of the parameters assessed, about 30% of the studies showed an unclear risk for incomplete outcome data (attrition bias) and 15% showed unclear risk for blinding of outcome assessment (detection bias) as seen in Fig. 2 and Fig. 3.
**Stefano Corbella (2021)(Corbella et al. 2021)**	**Risk of bias assessment -** Cochrane Handbook for Systematic Reviews<br><br>**Quality of evidence assessment -** Grading of Recommendations, Assessment, Development and Evaluations (GRADE)<br><br>**Assessment of publication bias -** Egger ́s bias.	**Risk of bias assessment -** The results of the risk of bias evaluation for studies involving healthy and systemically compro- mised subjects are shown in Appendix 3. Briefly, the evaluation of 17 out of 38 studies (43.6%) raised some con- cerns about the risk of bias, while the others (56.4%) were judged to be at low risk. The main concerns about the risk of bias evaluation were due to inadequate description of the randomization and allocation methods (16 of 38 studies, 41.0%), the number of dropouts (3 of 38 studies, 7.7%), and the absence of the placebo (8 of 38 studies, 20.5%), which might have influenced the awareness of the patient of their assigned intervention. Twenty of the included papers reported that they were supported in different forms (financial support, grant or the products) by manufacturers of the host modulators tested.<br><br>**Quality of evidence assessment -** Table 2. Overall, the GRADE assessment revealed that the quality of evidence in the investigated field is low or very low and such evaluation was

		mainly due to the significant heterogeneity among studies, differences in treatment protocols and risk of bias.
**Sze Nga Ho (2020)(Ho et al. 2020)**	**Assessment of Risk of Bias -** The Cochrane Handbook for Systematic Reviews of Interventions	**Risk of bias -** the risk of bias for each study is summarized in Appendix S2. Five studies were graded at low risk across all the categories. Five studies failed to report clearly if the blinding was not broken until the completion of data analysis. One study did not explicitly present the method of random sequence generation and allocation concealment, which might lead to selection bias. One study did not sufficiently describe the method of allocation concealment and the blinding of participants and personnel.
**Victor Haruo Matsubara (2016) (Matsubara et al. 2016)**	**Cochrane Collaboration for systematic reviews of interventions** (Risk of Bias)	Low/Unclear/High risk of bias<br>All studies had a random sequence generation, using computer-based randomization programs, block approach or tables. However, the allocation concealment was not totally clear in four studies [18,23,27,30]. The studies of Della Riccia et al. [27] and Shah et al. [23] also had an unclear risk of bias in blinding of patients, as well as the researchers, and outcome assessment. One study was classified as having an unclear risk of bias for the "incomplete outcome data" parameter [28], as the authors did not report the reason/s for excluding patients data from the analysis.<br>**Table 2 -** bias analysis per parameter.
**Wim Teughels (2011)(Teughels et al. 2011)**	-	-
**Zohaib Akram (2020)(Akram et al. 2020)**	**Risk of bias (across RCT's) -** modified recommendations of the Consolidated Standards of Reporting Trials statement.<br>**Risk of bias (individual studies) -** Cochrane Handbook for Systematic Reviews of Inter-ventions<br><br>**Grading of Recommendations Assessment, Development and Evaluation' (GRADE)**	**Risk of Bias**<br>The extracted data for risk of bias for each trial and their results are presented in Table 3. All selected studies were RCTs. However, three studies did not state anything about sequence generation, while two trials did not describe about allocation methods. Only six trials reported about methods of allocation concealment. Two trials were categorized as unclear because the method of allocation was not described. Periodontal examiners were deemed blinded in six studies and unclear in one study. All studies reported about patients completing clinical trials, and the details about dropouts either in the flow chart or main text except one study. Of all RCTs assessed, the overall risk of bias was considered high in five clinical trials.<br><br>**Grade (Evidence Profile)**<br>Table 4 shows a summary of the various factors used to rate the quality of evidence and strength of recommendations according to GRADE. Taken together, the strength of a recommendation based on the quality of the evidence emerging from this review is estimated to be moderate. Given that the effect is large, the direction of recommendation emerging from this systematic review is strong in favour of the use of probiotics (L. reuteri) in the treatment of gingivitis (Table 4).
**Zohre Gheisary (2022)(Gheisary et al. 2022)**	**Cochrane risk–of–bias assessment tool version 2 designed for randomized trials**	Using the Cochrane risk–of–bias assessment tool version 2, the majority of studies included were classified as having a low risk of bias (n = 38). Additionally, 13 studies were classified as having some concerns, and another 13 studies were classified as having a high risk of bias. The majority of concerns were due to questions related to the randomization process domain. There were minimal concerns regarding the missing outcome data domain. The results are presented in Supplemental Table S3.

Abdulkareem A, Abdulbaqi H, Gul S, Milward M, Chasib N, Alhashimi R. 2021. Classic vs. Novel antibacterial approaches for eradicating dental biofilm as adjunct to periodontal debridement: An evidence-based overview. Antibiotics (Basel). 11(1).

Akram Z, Shafqat SS, Aati S, Kujan O, Fawzy A. 2020. Clinical efficacy of probiotics in the treatment of gingivitis: A systematic review and meta-analysis. Aust Dent J. 65(1):12-20.

Arbildo-Vega HI, Panda S, Bal A, Mohanty R, Rendon-Alvarado A, Das AC, Cruzado-Oliva FH, Infantes-Ruiz ED, Manfredi B, Vasquez-Rodrigo H et al. 2021. Clinical effectiveness of lactobacillus reuteri in the treatment of peri-implant diseases: A systematic review and meta-analysis. J Biol Regul Homeost Agents. 35(2 Suppl. 1):79-88.

Barboza EP, Arriaga PC, Luz DP, Montez C, Vianna KC. 2020. Systematic review of the effect of probiotics on experimental gingivitis in humans. Braz Oral Res. 34:e031.

Barootchi S, Ravida A, Tavelli L, Wang HL. 2020. Nonsurgical treatment for peri-implant mucositis: A systematic review and meta-analysis. Int J Oral Implantol (Berl). 13(2):123-139.

Canut-Delgado N, Giovannoni L, Chimenos-Küstner E. 2021. Probiotics against periodontal disease: A systematic review. : Are probiotics a posible treatment of periodontitis?

Corbella S, Calciolari E, Alberti A, Donos N, Francetti L. 2021. Systematic review and meta-analysis on the adjunctive use of host immune modulators in non-surgical periodontal treatment in healthy and systemically compromised patients. Sci Rep. 11(1):12125.

Donos N, Calciolari E, Brusselaers N, Goldoni M, Bostanci N, Belibasakis GN. 2020. The adjunctive use of host modulators in non-surgical periodontal therapy. A systematic review of randomized, placebo-controlled clinical studies. J Clin Periodontol. 47 Suppl 22:199-238.

Gao J, Yu S, Zhu X, Yan Y, Zhang Y, Pei D. 2020. Does probiotic lactobacillus have an adjunctive effect in the nonsurgical treatment of peri-implant diseases? A systematic review and meta-analysis. J Evid Based Dent Pract. 20(1):101398.

Gheisary Z, Mahmood R, Harri Shivanantham A, Liu J, Lieffers JRL, Papagerakis P, Papagerakis S. 2022. The clinical, microbiological, and immunological effects of probiotic supplementation on prevention and treatment of periodontal diseases: A systematic review and meta-analysis. Nutrients. 14(5).

Gou H, Chen X, Li L, Wang X, Xu Y. 2020. Additional clinical benefits of probiotics as an adjunctive therapy to nonsurgical periodontal treatment of periodontitis: A systematic review and meta-analysis. BMC Oral Health - under review.

Gruner D, Paris S, Schwendicke F. 2016. Probiotics for managing caries and periodontitis: Systematic review and meta-analysis. J Dent. 48:16-25.

Hardan L, Bourgi R, Cuevas-Suarez CE, Flores-Rodriguez M, Omana-Covarrubias A, Nicastro M, Lazarescu F, Zarow M, Monteiro P, Jakubowicz N et al. 2022. The use of probiotics as adjuvant therapy of periodontal treatment: A systematic review and meta-analysis of clinical trials. Pharmaceutics. 14(5).

Ho SN, Acharya A, Sidharthan S, Li KY, Leung WK, McGrath C, Pelekos G. 2020. A systematic review and meta-analysis of clinical, immunological, and microbiological shift in periodontitis after nonsurgical periodontal therapy with adjunctive use of probiotics. J Evid Based Dent Pract. 20(1):101397.

Hu D, Zhong T, Dai Q. 2021. Clinical efficacy of probiotics as an adjunctive therapy to scaling and root planning in the management of periodontitis: A systematic review

and meta-analysis of randomized controlled trails. J Evid Based Dent Pract. 21(2):101547.

Ikram S, Hassan N, Raffat MA, Mirza S, Akram Z. 2018. Systematic review and meta-analysis of double-blind, placebo-controlled, randomized clinical trials using probiotics in chronic periodontitis. J Investig Clin Dent. 9(3):e12338.

Jayaram P, Chatterjee A, Raghunathan V. 2016. Probiotics in the treatment of periodontal disease: A systematic review. J Indian Soc Periodontol. 20(5):488-495.

Liu J, Liu Z, Huang J, Tao R. 2022. Effect of probiotics on gingival inflammation and oral microbiota: A meta-analysis. Oral Dis. 28(4):1058-1067.

Martin-Cabezas R, Davideau JL, Tenenbaum H, Huck O. 2016. Clinical efficacy of probiotics as an adjunctive therapy to non-surgical periodontal treatment of chronic periodontitis: A systematic review and meta-analysis. J Clin Periodontol. 43(6):520-530.

Matsubara VH, Bandara HM, Ishikawa KH, Mayer MP, Samaranayake LP. 2016. The role of probiotic bacteria in managing periodontal disease: A systematic review. Expert Rev Anti Infect Ther. 14(7):643-655.

Mishra S, Misra SR, Panda S, Mohanty N, Manfredi B, Parrini M, Giacomello MS, Mortellaro C, Greco Lucchina A, Annunziata M et al. 2021. Role of probiotics in adjunct to non-surgical periodontal therapy in patients with chronic periodontitis: A systematic review and meta-analysis. J Biol Regul Homeost Agents. 35(2 Suppl. 1):67-78.

Ng E, Tay JRH, Saffari SE, Lim LP, Chung KM, Ong MMA. 2022. Adjunctive probiotics after periodontal debridement versus placebo: A systematic review and meta-analysis. Acta Odontol Scand. 80(2):81-90.

Priyanka K, kudlure SM, Chandra V, Reddy S, Kumar RVSK, Gomasani S. 2016. Probiotics in treatment and prevention of periodontal diseases: A systemic review. International Archives of BioMedical and Clinical Research. 2(3):7-12.

Saïz P, Taveira N, Alves R. 2021. Probiotics in oral health and disease: A systematic review. Applied Sciences. 11(17):8070.

Sayardoust S, Johansson A, Jonsson D. 2022. Do probiotics cause a shift in the microbiota of dental implants-a systematic review and meta-analysis. Front Cell Infect Microbiol. 12:823985.

Seminario-Amez M, Lopez-Lopez J, Estrugo-Devesa A, Ayuso-Montero R, Jane-Salas E. 2017. Probiotics and oral health: A systematic review. Med Oral Patol Oral Cir Bucal. 22(3):e282-e288.

Silva AP, Cordeiro TO, da Costa RA, Martins A, Dantas EM, Gurgel BCV, Lins R. 2020. Effect of adjunctive probiotic therapy on the treatment of peri-implant diseases - a systematic review. J Int Acad Periodontol. 22(3):137-145.

Song D, Liu XR. 2020. Role of probiotics containing lactobacillus reuteri in adjunct to scaling and root planing for management of patients with chronic periodontitis: A meta-analysis. Eur Rev Med Pharmacol Sci. 24(8):4495-4505.

Teughels W, Loozen G, Quirynen M. 2011. Do probiotics offer opportunities to manipulate the periodontal oral microbiota? J Clin Periodontol. 38 Suppl 11:159-177.

Vives-Soler A, Chimenos-Kustner E. 2020. Effect of probiotics as a complement to non-surgical periodontal therapy in chronic periodontitis: A systematic review. Med Oral Patol Oral Cir Bucal. 25(2):e161-e167.

Yanine N, Araya I, Brignardello-Petersen R, Carrasco-Labra A, Gonzalez A, Preciado A, Villanueva J, Sanz M, Martin C. 2013. Effects of probiotics in periodontal diseases: A systematic review. Clin Oral Investig. 17(7):1627-1634.
